# Maternal and child characteristics and health practices affecting under-five mortality: A matched case control study in Gamo Gofa Zone, Southern Ethiopia

**DOI:** 10.1371/journal.pone.0202124

**Published:** 2018-08-15

**Authors:** Girma Temam Shifa, Ahmed Ali Ahmed, Alemayehu Worku Yalew

**Affiliations:** School of Public Health, Addis Ababa University, Addis Ababa, Ethiopia; College of Medicine, University of Ibadan, NIGERIA

## Abstract

**Background:**

Though Ethiopia has shown a considerable improvement in reducing under-five mortality rate since 1990, many children still continue to die prematurely. Mixed results have been reported about determinants of under-five mortality. Besides, there is paucity of mortality studies in the current study site. Therefore, this study was conducted to assess maternal and child health related predictors of under-five mortality in Southern Ethiopia.

**Methods:**

A matched case control study was conducted in 2014 in Arba Minch Town and Arba Minch Zuria District of Gamo Gofa Zone, Southern Ethiopia. Conditional logistic regression was employed to identify the predictors of under-five mortality. Sampling weight was applied to account for the non-proportional allocation of sample to different clusters. Based on the Mosley & Chen's analytical framework for under-five and infant mortalities, the predictors were organized in to three groups: 1) personal illness control, 2) child feeding and newborn care and 3) other maternal and child related factors.

**Results:**

Among personal illness control related factors: lack of post-natal care, immunization status of the child and lack of Vitamin A supplementation were significantly associated with higher rate of under-five mortality. Not breastfeeding and delaying first bath at least for 24 hours were child feeding and newborn care related factors which were found to be significantly associated with under-five mortality. Among other maternal and child related factors, shorter previous birth interval, history of death of index child’s older sibling, being multiple birth and live birth after the index child were significantly associated with under-five mortality.

**Conclusions:**

In order to maintain reduction of under-five mortality during the Sustainable Development Goals era, strengthening of maternal and child health interventions, such as post-natal care, family planning, immunization, supplementation of Vitamin A for children older than six months, breastfeeding and delaying of first bath after delivery at least for 24 hours are recommended.

## Background

In Ethiopia, both under-five and infant mortality rates significantly declined during the past decades [1], this might be as a result of the different health initiatives, such as expansion of health extension program in the country during this period [2]. However, child mortality rates in the country are among the highest in the world and about one in every 21 and 15 Ethiopian children dies before his/her first and fifth birthday, respectively [3]. Under-five mortality rate in Southern Nations, Nationalities and People’s Region (SNNPR), where the study conducted is among the highest in the country [1].

Many factors have been implicated for high under-five mortality in developing countries in general and in Ethiopia in particular. For example, children are thought to be dying owing to deliveries attended by untrained traditional birth attendants, relatives and neighbors at home [1], a practice that may present risks to both the mother and the newborn. Even though some are skeptical about the association between use of maternal and child health services and survival in developing countries, partly because of lack of health care service quality [4, 5], many studies support the positive association between health care service use, such as institutional delivery and maternal and child mortality [6, 7]. Place of delivery is also found to be associated with early childhood illnesses such as neonatal tetanus [8]. However, protective effect of institutional delivery on under-five mortality was not demonstrated by studies conducted in Ethiopia [9, 10]. It is possible to argue that this lack of association may be as a result of limited quality of health care services, in many developing countries which remains questionable [4, 5].

As a strategy for improved maternal and child survival, the World Health Organization (WHO) recommends a pregnant mother to attend at least four prenatal visits during her pregnancy [11]. This was supported by studies, that demonstrated the significant effect of antenatal care (ANC) service utilization on survival of children [6, 12]. However, effects of ANC and institutional delivery services may vary depending on the level of quality of services being provided.

Immunization may be one of the effective child-survival interventions to date as it addresses diseases which contribute significantly to high under-five mortality; including neonatal tetanus, whooping cough, polio and measles [13]. Immunization status of a child was shown to be an important factor for under-five mortality [7]. However, such conclusions were not made by other studies [14, 15]. Besides, the effect was shown to be higher in communities with low socioeconomic status [16], indicating that its effect is context dependent.

In addition to immunization, Vitamin A supplementation is one of the health interventions which are expected to have many health benefits for children. Vitamin A status of children aged six months to five years was shown to be an important predictor of mortality [17, 18]. The effect was significant, especially in populations with low prevalence of clinical signs of Vitamin A deficiency [18]. As Vitamin A was shown to significantly reduce deaths attributed to diarrhea and measles [17, 18], it is natural to expect its effect to be dependent on the prevalence of these causes of mortality. Insignificant association between Vitamin A and under-five mortality was also observed by another study [19].

Promotion of breastfeeding is considered to be a key component of child survival strategies in many developing countries [20]. Accordingly, exclusive breastfeeding during the first six months of life, early initiation of breastfeeding within one hour of birth and continuation of breastfeeding with other complementary foods till 2^nd^ birth day of the child are recommended [21, 22]. As exclusive breastfeeding is reported to be associated with intermediate causes of under-five mortality, such as diarrhea and respiratory infections [23, 24], its effect on under-five mortality may vary based on the prevalence of these intermediate causes. Besides, the extent of the effect of breastfeeding on child mortality varies across the age category of children, with younger infants deriving the greatest benefit [25]. There are, however, findings which are inconsistent with the above conclusions, showing insignificant association between child mortality and breastfeeding [26]. Besides, problems with breastfeeding, which are proxy indicators of exclusive breastfeeding, were not shown to have significant effect on under-five mortality in Africa [15, 27].

WHO recommends hygiene during delivery, keeping the newborn warm, early initiation of breastfeeding, care of the eyes, care during illness, immunization and care of low-birth-weight newborns as essential newborn care practices [28]. However, majority of deliveries in developing countries in general and in Ethiopia in particular take place at home by untrained birth attendants [1]. As a result, many lifesaving practices are either missed or done inappropriately, contributing to widespread maternal and childhood morbidities and mortalities. The effect of the different interventions recommended during pregnancy and childbirth, however, is expected to be dependent on how they are brought to the users [29].

Other maternal and child characteristics have also been studied and attributed to child and maternal deaths. For example, mothers with short birth intervals may have insufficient time to restore their nutritional reserves, a situation which is thought to adversely affect fetal growth. As a result, WHO recommends at least 24 months of gap between a birth and a subsequent pregnancy [30]. This was also supported by other studies [31, 32]. However, it is argued that, short birth intervals can be a consequence than a cause of child mortality [33], i.e., parents with child loss are more likely to have next child sooner than those with alive child.

It has been reported that, first born children are less likely to die than higher birth order children, possibly as a result of firstborns being favored with vital resources such as food and care [34] and this is supported by other studies [32, 35]. To the contrary, it is argued that first born children are more likely to be born to mothers at younger ages thereby experiencing a higher mortality risk [14]. Insignificant association between child mortality and birth order was reported by another study too [26].

In addition to the inconsistent findings of studies outlined above, the effect of different maternal and child related factors on under-five mortality may vary through time because of changes in the life style of nations. Furthermore, the associations may be context dependent. Mortality studies in the study site have been lacking, as the Arba Minch Demographic Surveillance Site (DSS), which is located at the extreme south of the country, is relatively recently established research center in the area. These are the reasons that call for investigation of determinants in general and maternal and child related factors in particular of under-five mortality in the study area. Therefore, this study investigated maternal and child related predictors of under-five mortality in Gamo Gofa Zone, southern Ethiopia by including data from both DSS and non-DSS kebeles.

Outputs of the study will enhance comprehensive knowledge about determinants of under-five mortality in the country by providing information from those uninvestigated areas with a wider population category. The study tried to identify contextualized and prioritized maternal and child health related areas in order to sustain reduction of under-five mortality and improve child survival during the Sustainable Development Goals (SDGs) era. The determinant factors for this study were framed using Henry Mosley and Lincoln Chen’s analytic framework for the study of determinants of under-five mortality. The adapted conceptual framework for the overall determinants of under-five mortality is presented elsewhere [36].

## Methods

### Study area

The study was conducted in Arba Minch Zuria District and Arba Minch Town of Gamo Gofa Zone, which has 15 districts (woredas) and two town administrations standing at an altitude ranging from 600 to 3300 meters above sea level. Arba Minch Town, capital of the zone is 502km south of Addis Ababa (capital city of Ethiopia). In 2014, the total population of the zone was projected to be 1,901,953 (with 285,043 Urban (15%) and 1,616,910 Rural (85%) residents) [37]. There were three hospitals and 68 health centers serving the population of the zone during the study period. The study area is known for its banana, apple and fish production which may impact child nutrition thereby child survival. Arba Minch Zuria District has 29 kebeles (lowest administrative units in Ethiopia) with three different climatic/agro-ecological zones; high land (Dega), mid land (Weinadeaga) and low land (Kola), which is useful for representing population of different agro-ecologies. The total population of the district was projected to be 185,302 in 2014. Arba Minch Town is included to represent the urban population of the zone. The total population of the town was projected to be 135,452 [37]. The town was divided in to 11 urban kebeles. Arba Minch DSS was established in 2009 in nine kebeles of the Arba Minch Zuria District. Details of the study area are presented elsewhere [38].

### Study design and period

A matched case control study design was implemented in 2014.

### Source and study population

The source population was all under-five children in the study area. The study population consisted of cases of children who died between March 01, 2011 and September 30, 2014 and randomly selected two live controls of under-five children born within one-month time in the same locality with each case.

### Inclusion and exclusion criteria

Only live births were included i.e. stillbirths were excluded.

### Sample size determination

The sample size was calculated using Statcalc command of Epi info 7 statistical software package. The prevalence of exposure (selected determinants) among controls and cases were estimated from previous studies [9, 10, 39–42] or calculated from the data available from Arba Minch DSS’s Database. Then, the sample size required for detecting an odds ratio (OR) with a two-sided probability of type I error of 5% and a power of 80% was determined for main exposure variables with control to case ratio of two. The maximum sample size, which corresponded to home delivery was taken, subsequently. As depicted in Table 1, a minimum of 241 cases and 482 controls were required. By applying a design effect of 1.5 and adding 5% to compensate for non-response, a total of 380 cases and 760 controls were required. The details of the estimations, indicators and sample size required for selected variables are summarized in Table 1.

**Table 1:** Variables and their corresponding indicators used for sample size estimation, Gamo Gofa Zone, 2014.

Determinant/exposure variables	% of control exposed	%cases exposed	Confidence level	Power	No. cases	No. controls	Total
Lack of antenatal care (ANC)	20.6	36.5	95%	80%	92	184	276
Home delivery	87.2	93.8	95%	80%	241	482	723
Lack of vaccination	27	66	95%	80%	18	36	54
Parity> = 5	23.6	36.7	95%	80%	141	282	423
Birth interval<24m	13.3	28.8	95%	80%	78	155	233
No Exclusive breastfeeding	14.1	28.1	95%	80%	96	192	288

### Sampling technique

Arba Minch Town and the Arba Minch Zuria District were purposively selected. Then, all 11 kebeles of Arba Minch Town and nine kebeles of the Arba Minch DSS (initially they were randomly selected out of 29 kebeles of Arba Minch Zuria District) were taken. Additionally, 11 non-DSS kebeles from Arba Minch Zuria District were randomly selected. Then, a census of the 11 non-DSS kebeles of the Arba Minch Zuria District and 11 kebeles of Arba Minch Town was conducted. The census identified all children who were born between September 01, 2007 and September 30, 2014 and under five children who died between March 01, 2011 and September 30, 2014 in those kebeles. As the Arba Minch DSS has been tracking all births and deaths since its establishment, children born between August 01, 2009 and September 30, 2014 and under five children who died between March 01, 2011 and September 30, 2014 in Arba Minch DSS kebeles were tracked from its database. Further detail of this part is presented elsewhere [38]. From the census and the Arba Minch DSS, 383 cases that fulfilled the inclusion criteria were identified. And all of them with their corresponding matched and randomly selected 766 controls were approached as that number was almost equal to the sample size estimated above.

Specifically, all under-five children who died between March 01, 2011 and September 30, 2014 were classified as cases and for each case two alive children who were matched for date of birth (born within one month) and lived in the same kebele as the case were selected. To minimize control selection bias, the two controls were selected randomly after identifying all potential controls for a given case.

Data from 381 cases and 762 controls were obtained. Data from two cases and their corresponding controls were not retrieved from two kebeles, since they migrated out of their initial location.

### Data collection

A pre-tested closed ended structured Amharic (local language) questionnaire was utilized for data collection (S1 Appendix). The questionnaire was developed in English based on literatures and translated in to Amharic, then back translated to English to ensure for its consistency. Finally, the Amharic version was used for data collection among both cases and controls. Two data collectors (at least certificate holders after completing grade 10) per kebele were recruited and trained on data collection procedure. Four supervisors who were master’s degree holders supervised the data collection process. The principal investigator/author strictly followed the data collection process.

### Measurements and operational definitions

#### Dependent variable

Mortality (dead or alive) status of the index child.

#### Independent variables

Socioeconomic and demographic characteristics (the results are presented elsewhere [36]), personal illness control related variables, child feeding and newborn care related variables and other maternal and child related variables.

#### Wealth index and maternal decision status

were computed using principal component analysis of reported ownership of household assets and maternal decision related variables, respectively (the details of the variables and assumptions are presented elsewhere [36]).

#### Ever breastfeeding, exclusive breastfeeding and initiation of breastfeeding

were ascertained by asking the respondents to recall their experiences since birth of the index child. This was the only option that could be applied specially for cases (for the details see the questionnaire (S1 Appendix)).

#### Immunization and Vitamin-A supplementation status

were determined mainly by using immunization card. In the absence of immunization card, the respondents were asked to recall the type of vaccine/medicine the child had taken during the latest vaccination.

#### Infant mortality

is the probability of dying between birth and the first birthday.

#### Under five-mortality

is the probability of dying before the fifth birthday.

### Data processing and management

Data were entered into Epi info by two trained data encoders. The data were edited, coded, and cleaned using Epi Info Version 3.5.1. The analysis was performed by STATA version 11. Data entry was strictly followed and checked by the principal investigator on daily basis. Double entry of 10% of the questionnaires was made.

### Data analysis

To identify maternal and child health related predictors of under-five mortality, conditional logistic regression was performed. Sampling weight was employed in the analysis to account for the non-proportional allocation of the sample. The sampling weight was taken as the reciprocal of probabilities of selection. The probability of selection for urban was = 1/2*1*306/5745 = 0.027 (as one out of 2 urban Districts and all the kebeles in selected District were included. Three hundred six out of 5745 children identified by the census were included). The corresponding sampling weight for urban was calculated to be 37.6. For that of the rural = 1/15*20/29*837/14416 = 0.0027(as one out of 15 rural districts, twenty out of 29 kebeles of the District were included and 837 out of 14416 children identified were included in the study). The corresponding sampling weight for rural was calculated to be 374.6.

The models were fitted based on Mosley & Chen's analytical framework for the study of determinants of child mortality [43]. According to this framework, distal factors operate through proximate factors, such as personal illness control related, child feeding and newborn related and other maternal and child related factors, to affect under-five mortality. The framework classifies distal factors into three levels (individual, household and community). Accordingly, first a model for distal factors was fitted (the findings for distal factors are presented elsewhere [36]). As community level factors were controlled at design stage by matching of the two groups (cases and controls), we only included individual and household level distal factors in the models. Then, those distal factors which were significant at p-value of 0.1 were controlled for models of proximate variables. Accordingly, three separate models were fitted for proximate factors (personal illness control related variables, child feeding and newborn related variables and maternal and child related variables) by controlling distal factors which were significant at p-value of 0.1. This strategy of modeling for a specific category (relatively less variables per category) allowed inclusion of all relevant variables for a given proximal category which were selected based on literature. Sex of the child was also included in all models, as it was found to be significantly associated with under-five mortality in previous paper [38]. Though the main focus of this study was under-five mortality, as a sub analysis, the same modeling strategies were replicated and presented for infant mortality.

As described in detail elsewhere [36], model diagnostic was conducted following each model using different STATA commands. Presence of interaction/effect modification was also assessed for suspected variables. No significant interaction/ effect modification was observed. Collin command was used to assess presence of multicollinearity among predictors. Tolerance value < = 0.1 or variance inflation factor (VIF) of greater than or equal to 10 were taken as an indicator of presence of multicollinearity. No multicollinearity was identified as all VIFs were less than 5.

### Ethical considerations

Ethical clearance and approval was obtained from the Institutional Review Board of the College of Health Sciences at Addis Ababa University. Letters were written to all concerned bodies and permissions were secured at all levels. After explaining the purpose of the study and confidentiality of the data, verbal informed consent was obtained from each respondent. A trained interviewer sat down with each respondent and went through the consent form in a private space, answered any questions the respondent might have and secured his/her consent. A waiver of written consent from the IRB was sought and secured since this might be the only form of identifier. The main risk to participants might be breach of confidentiality. The confidentiality of the information was assured through anonymous interviews and securing the collected data in a locked cabinet. Besides, only aggregated data were reported so that no information could be linked to individuals (this detail is also presented elsewhere [36]).

## Results

Data from 381 cases and 762 controls were retrieved giving a response rate of 100% (against the estimated sample). Majority 837(73.2%) of the children were from rural kebeles. Slightly more than half 210(55.1%) of the cases and 382(50.1%) of the controls were males. With regard to maternal education, more than half 210(55.1%) of the cases and less than half 362(47.5%) of the controls had no formal education. Pertaining to ethnic background, majority of mothers of the cases 286(75.1%) and the controls 571(74.9%) were Gamo. Most mothers of the cases 264(69.3%) and controls 539(70.7%) were housewives. Nearly half of husbands of the cases 191(50.1%) and controls 433(56.8%) were farmers. Using wealth index, 124(32.5%) of the cases and 328(43.0%) of the controls were classified as rich. Majority of the cases 262(68.8%) and controls 492(64.6%) were born at home. The details of the socio-demographic characteristics of the study participants were presented elsewhere [36].

### Association between personal illness control related factors and under-five mortality

Among factors classified as ‘personal illness control’; post-natal care (PNC) for the index pregnancy/child, antenatal care (ANC) for the index pregnancy, immunization status of the child for his /her age and whether the child had Vitamin A supplementation or not were significantly associated with under-five and/or infant mortality (Table 2).

**Table 2:** The association between personal illness control related factors and under-five mortality, Gamo Gofa Zone, 2014.

Characteristics	Under-five					Infants				
	Controls N(%)	Cases N(%)	AOR^a^	[95% Conf. Interval]		Controls N(%)	Cases N(%)	AOR^b^	[95% Conf. Interval]	
**Had post-natal care for the index child**										
Yes	260(34.1)	99(26.0)	Ref^c^			187(35.6)	74(28.1)	Ref		
No	502(65.9)	282(74.0)	**2.27**	**1.25**	**4.11**	339(64.5)	189(71.9)	**2.35**	**1.02**	**5.45**
**Number of ANC follow up for index pregnancy**										
No ANC follow up	97(12.7)	68(17.9)	Ref			174(33.1)	76(28.9)	Ref		
Less than 4	245(32.2)	113(29.7)	0.88	0.51	1.54	174(33.1)	76(28.9)	0.58	0.27	1.24
Greater than or equal to 4	420(55.1)	200(52.5)	0.72	0.42	1.24	287(54.6)	134(51.0)	**0.45**	**0.23**	**0.90**
**Place of delivery**										
Home	488(64.0)	261(68.5)	Ref			322(61.2)	166(63.1)	Ref		
Hospital	117(15.4)	53(13.9)	1.43	0.32	6.45	89(16.9)	47(17.9)	1.76	0.27	11.53
Health center	126(16.5)	54(14.2)	0.96	0.20	4.62	93(17.7)	41(15.6)	0.95	0.13	7.00
Health post	21(2.8)	10(2.6)	1.72	0.72	4.15	13(2.5)	6(2.3)	2.24	0.66	7.56
Others	10(1.3)	3(0.8)	0.58	0.09	3.86	9(1.7)	3(1.1)	0.62	0.08	4.87
**Attendant of delivery**										
Skilled health professionals	250(32.8)	114(29.9)	Ref			188(35.7)	94(35.7)	Ref		
Health extension worker	49(6.4)	18(4.7)	0.64	0.14	2.88	36(6.8)	9(3.4)	0.37	0.05	2.74
TBA	57(7.5)	31(8.1)	0.36	0.07	1.90	32(6.1)	17(6.5)	0.47	0.05	4.05
Relative/Neighbor/Mother Herself	406(53.3)	218(57.2)	0.61	0.14	2.81	270(51.3)	143(54.4)	0.46	0.07	3.17
**Immunization status of the child**										
Fully immunized for age	435(77.4)	137(48.8)	Ref			241(73.9)	58(35.6)	Ref		
Partially immunized for age	80(14.2)	68(24.2)	**3.63**	**2.02**	**6.51**	52(16.0)	43(26.4)	**4.10**	**1.81**	**9.30**
Not immunized	47(8.4)	76(27.0)	**11.04**	**5.17**	**23.59**	33(10.1)	62(38.0)	**18.54**	**6.22**	**55.26**
**Did the child have vit. A (for those > = 6Month old)**										
Yes	581(76.3)	155(40.7)	Ref			397(75.5)	77(29.3)	Ref		
No	181(23.8)	226(59.3)	**7.60**	**4.72**	**12.23**	129(24.5)	186(70.7)	**15.29**	**6.38**	**36.65**

^a^Besides variables in the table, adjusted for sex of the child, mother’s education, wealth index, husband occupation and marital status of the mother

^b^Besides variables in the table, adjusted for sex of the child, mother’s education, wealth index, husband occupation and marital status of the mother

^c^reference value

The odds of mortality was about two times higher among under-five children who lacked PNC (AOR of 2.27(1.25–4.11)) than those who had. Similarly, lack of PNC service was shown to increase the odds of mortality by about 2 fold during the first year of life of the child (AOR of 2.35(1.02–5.45)). Infants who had at least four ANC follow-up during their pregnancy had less odds of mortality (AOR of 0.45(0.23–0.90)) than those infants who lacked ANC. Though it was not significant, similar effect was observed among under-five children with AOR of 0.72(0.42–1.24) (Table 2).

The odds of mortality among under-five children who were partially immunized for their age, was about four times higher (AOR of 3.62(2.02–6.50)) than those fully immunized. The odds of mortality among under-five children, who were not immunized at all, was about 11 times higher (AOR of 11.02(5.16–23.53)) than children who were fully immunized for their age. Similarly, significant increment of odds of mortality was observed among partially (AOR of 4.10(1.81–9.30)) and not immunized (AOR of 18.54(6.22–55.26)) infants than fully immunized infants (Table 2).

Under-five children who did not have Vitamin A supplementation at least once after six months of their age had about 8 times (AOR of 7.61(4.72–12.26)) higher odds of mortality than those who did. Similarly, lack of Vitamin A supplementation was shown to increase odds of mortality during the first year of age of the child (AOR of 15.29(6.38–36.65)). In this study, place or attendants of delivery of index child were not significantly associated with under-five mortality (Table 2).

### Association between child feeding and newborn care practices and under-five mortality

Among factors related with child feeding and newborn care practices, history of ever breastfeeding and timing of first bath of the index child were significantly associated with under-five mortality. Under-five children who were never breastfed had about 8 times (AOR of 8.09(4.08–16.05)) higher rate of odds of mortality than those who were ever breastfed. Similarly, those infants who were never breastfed had higher rate of odds of mortality (AOR of 14.19(5.51–36.50)) than those who were ever breastfed. Delaying first bath of a child, at least, to 24 hours after birth was shown to reduce odds of under-five mortality by 50% (AOR of 0.50(0.34–0.73)) and odds of infant mortality by 54% (AOR of 0.46(0.28–0.77)) than having first bath within 24 hours of birth (Table 3).

**Table 3:** The association between child feeding and newborn care practices and under-five mortality, Gamo Gofa Zone, 2014.

Characteristics	Under-five					Infants				
	Controls N(%)	Cases N(%)	AOR^a^	[95% Conf.Interval]		Controls N(%)	Cases N(%)	AOR^b^	[95% Conf.Interval]	
**Ever Breast fed**										
Yes	742(97.4)	311(81.6)	Ref^c^			513(97.5)	193(73.4)	Ref		
No	20(2.6)	70(18.4)	**8.09**	**4.08**	**16.05**	13(2.5)	70(26.6)	**14.19**	**5.51**	**36.50**
**First breastfeeding started**										
Within 1hr	638(86.0)	249(80.1)	Ref			435(84.8)	149(77.2)	Ref		
After 1hr	104(14.0)	62(19.9)	1.55	0.95	2.51	78(15.2)	44(22.8)	1.43	0.80	2.59
**Breastfeeding status within 6month of age**										
Exclusive	424(57.1)	153(49.2)	Ref			303(59.1)	106(54.9)	Ref		
Predominantly	37(5.0)	10(3.2)	0.94	0.38	2.29	23(4.5)	9(4.7)	1.19	0.41	3.45
Partially	281(37.9)	148(47.6)	1.29	0.86	1.94	187(36.5)	78(40.4)	0.91	0.54	1.51
**Bottle feeding**										
Yes	79(14.1)	41(14.6)	Ref			49(15.0)	22(13.5)	Ref		
No	483(85.9)	240(85.4)	0.89	0.49	1.64	277(85.0)	141(86.5)	1.41	0.57	3.50
**Timing of first bath**										
Within 24 hour of birth	429(56.3)	268(70.3)	Ref			296(56.3)	195(74.1)	Ref		
After 24 hour of birth	333(43.7)	113(29.7)	**0.50**	**0.34**	**0.73**	230(43.7)	68(25.9)	**0.46**	**0.28**	**0.77**
**Anything applied to umbilical wound**										
Yes	215(28.2)	108(28.4)	Ref			146(27.8)	69(26.2)	Ref		
No	547(71.8)	273(71.7)	0.91	0.63	1.32	380(72.2)	194(73.8)	0.88	0.53	1.44

^a^Besides variables in the table, adjusted for sex of the child, mother’s education, wealth index, husband occupation and marital status of the mother

^b^Besides the variables in the table, adjusted for sex of the child, mother’s education, wealth index, marital status of the mother

^c^reference value

Although the associations were in expected directions, associations between under-five and/or infant mortality and factors like exclusive breastfeeding status of the child, history of bottle feeding, breastfeeding initiation time and history of something applied to the umbilical wound were not statistically significant (Table 3).

### Association between other maternal and child related factors and under-five mortality

Among other maternal and child related factors included in model 3 (Table 4); birth interval, history of child death before the index child, type of birth of index child and presence of live birth after index child were significantly associated with under-five and/or infant mortality. Under-five children who had birth interval of 24–36 months (AOR of 0.48(0.28–0.82)) or more than 36 months (AOR of 0.46(0.26–0.79)) had less odds of mortality than those children who had birth interval of less than 24 months. Similarly, infants who had birth interval of 24–36 months (AOR of 0.28 (0.14–0.54)) or more than 36 months (AOR of 0.29 (0.15–0.55)) had less odds of mortality than infants who had birth interval of less than 24 months (Table 4).

**Table 4:** The association between maternal and child factors and under-five mortality, Gamo Gofa Zone, 2014.

Characteristics	Under-five					Infants				
	Controls N(%)	Cases N(%)	AOR^a^	[95% Conf. Interval]		Controls N(%)	Cases N(%)	AOR^b^	[95% Conf. Interval]	
**Age of mother at child birth**			1.03	0.97	1.09			1.06	1.00	1.13
**Number of pregnancies before index child**			1.04	0.87	1.25			0.91	0.72	1.15
**Birth order of index child**										
first	200(26.3)	103(27.0)	Ref^c^			138(63.6)	9(36.4)	Ref		
second	147(19.3)	63(16.5)	0.71	0.13	3.76	103(68.2)	48(31.8)	5.29	0.47	59.54
Third	144(18.9)	85(22.3)	1.06	0.20	5.32	109(69.4)	48(30.6)	3.73	0.40	34.88
fourth	271(35.6)	130(34.1)	0.71	0.14	3.19	176(66.7)	88(33.3)	4.35	0.47	40.03
**Birth Interval**										
<24months	233(30.6)	155(40.7)	Ref			156(29.7)	106(40.3)	Ref		
24-36months	157(20.6)	69(18.1)	**0.48**	**0.28**	**0.82**	99(18.8)	40(15.2)	**0.28**	**0.14**	**0.54**
>36months	175(23.0)	55(14.4)	**0.46**	**0.26**	**0.79**	136(25.9)	39(14.8)	**0.29**	**0.15**	**0.55**
first birth	197(25.9)	102(26.8)	1.01	0.16	6.45	135(25.7)	78(29.7)	0.64	0.07	5.67
**History of child death before index child**										
No	486(86.5)	218(78.4)	Ref			346(89.2)	146(79.3)	Ref		
Yes	76(13.5)	60(21.6)	**1.97**	**1.07**	**3.61**	42(10.8)	38(20.7)	**3.37**	**1.51**	**7.50**
**Type of birth**										
Single birth	752(98.7)	347(91.1)	Ref			520(98.9)	235(89.4)	Ref		
Multiple birth	10(1.3)	34(8.9)	**13.72**	**5.26**	**35.79**	6(1.1)	28(10.7)	**21.18**	**5.85**	**76.74**
**History of child birth after index child**										
No	619(81.2)	212(55.6)	Ref			**n/a**	**n/a**			
Yes	143(18.8)	169(44.4)	**5.06**	**2.80**	**9.16**	**n/a**	**n/a**			

^a^Besides variables in the table, adjusted for sex of the child, mother’s education, wealth index, husband occupation, marital status of the mother

^b^Besides variables in the table, adjusted for sex of the child, mother’s education, wealth index, marital status of the mother

^c^reference value

n/a-not applicable

Under-five children (AOR of 1.97(1.07–3.61)) and infants (AOR of 3.37 (1.51–7.50)) with history of death of older siblings had higher rate of odds of mortality than those without history of death of older siblings (Table 4).

Those under-five children whose births were multiple had higher rate of odds of mortality (AOR of 13.72(5.26–35.79)) than those who were singleton birth. Similar effect was observed among infants (AOR of 21.18 (5.85–76.74)). Under-five children of mothers with history of birth after the index child had a higher rate of mortality (AOR of 5.06(2.80–9.16)) than those without. However, age of mothers at child birth, gravidity and birth order of index child were not statistically significantly associated with under-five mortality in this study (Table 4).

## Discussion

This study identified vital maternal and child health related predictors of under-five mortality by controlling for effects of potential confounders at design and analysis stage. To enhance that, efforts such as matching at design stage and weighted conditional logistic regression during analysis were employed. Lack of post-natal care, immunization status of the child, lack of Vitamin A supplementation, lack of breastfeeding, delaying first bath at least for 24 hours, shorter previous birth interval, history of death of older sibling of the index child, multiple birth and live birth after the index child were determinant factors which were significantly associated with under-five mortality.

However, as the information was collected retrospectively; systematic errors, such as recall bias might have affected some of the responses. Besides, because of the wide confidence interval of the effect sizes of some of the variables, which was attributed to small sample size, findings particularly those related to determinants of infant mortality need to be interpreted cautiously. In controlling confounding factors, the study may not be exhaustive enough to address all confounding factors. However, it is possible to assume that the confounding effect of other proximate variables were at least partially addressed by controlling distal factors, which are assumed to be operating through those proximate factors.

Both mothers and their newborns are vulnerable during the postnatal period and provision of PNC services at this period is expected to prevent maternal and child morbidity and mortality [44, 45]. This time is crucial not only to treat complications arising from the delivery, but also to provide women with important information on how to care for themselves and their children. In support of this, in the current study, lack of PNC was found to increase the odds of mortality by about 2.3 fold during the first five years of age and by about 2.4 fold during the first year of age of the child. This finding is in line with another study [46] which analyzed Demographic and Health Survey data from sub-Saharan African countries. Those findings call for strengthening of provision of PNC as one of the main strategies for under-five mortality reduction during the SDGs era.

Infants whose mothers had at least four ANC follow-up during their pregnancy had less odds of mortality than those infants who lacked ANC. Though it was not significant, similar effect of ANC was observed among under-five children. Similarly despite not being statistically significant, experience of even less than four ANC follow ups during pregnancy of the index child was shown to reduce odds of under-five and infant mortality. Similar protective effect of ANC follow up was observed by other studies [6, 12]. This may be because of the criticality of this time for the mother and the fetus helping to identify high risk pregnancy including multiple pregnancy (which was shown to be a risk factor in this study) in order to take appropriate actions.

In contrast to reports of other previous studies [6, 7] about positive effect of institutional or skilled attendance of delivery on under-five mortality, place and attendance of delivery of the index child were not significantly associated with under-five mortality in the current study. Nevertheless, institutional delivery did not show a protective effect in other studies conduct in Ethiopia [9, 10]. Lack of significant association between delivery at hospital or skilled attendant and neonatal mortality was also reported by another study [47]. This may be due to the fact that complicated cases were more likely to come for institutional delivery than to stay at home. However, health service delivery systems in developing countries are criticized for failing to bring the expected health benefits at expected level, partly because of dysfunctional health services [4, 5]. So, such findings could be motivating factors to question and investigate the quality of delivery services provided by health facilities in the study areas.

Immunization is the most effective child-survival intervention to date and the immunization status of children was reported to be an important factor of survival [7] with its greater impact in communities with low socioeconomic status [16]. Similarly, in the current study, immunization status for age of the child was significantly associated with under-five and infant mortality. The odds of under-five and infant mortality were also high among children who lacked Vitamin A supplementation at least once after six months of age than those who took. Vitamin A supplementation is another child health intervention and reported to reduce under-five mortality attributed to diarrhea and measles [17, 18]. Both the above findings ratify the importance of strengthening of these interventions for sustenance of the reduction of under-five mortality and achieving targets of child health related SDGs.

Among factors related to child feeding and newborn care practices, history of ever breastfeeding was found to be significantly associated with low under-five and infant mortality. Breastfeeding has been reported to be a major determinant of child health and mortality [42, 48]. As a result, the promotion of breastfeeding is considered to be a key component of child survival strategies in many developing countries [20]. Although, owing to the design of this study, problem of residual reverse causality could not be ruled out, the finding of this study support the promotion of breastfeeding as a strategy for better child survival in developing countries like Ethiopia.

In this study, exclusive breastfeeding status of the child and history of bottle feeding were not significantly associated with under-five mortality. This was in line with the finding of another study [49]. Insignificant difference of mortality among exclusively breast fed children and pre-dominantly breast fed children was also reported by another study [48]. Problem of breastfeeding which may be a proxy indicator of lack of exclusive breastfeeding was also not associated with under-five mortality by another study [50]. The lack of significant association in the current study may be because of the rareness of these practices in the study area, as exclusive breastfeeding is not widely practiced, that only 32% of Ethiopian infants 4–5 months were reported to be exclusively breast fed [1].

Delaying first bath of a child at least to 24 hours after birth was shown to reduce odds of under-five and infant mortality. This finding reinforces the WHO recommendation of delaying first bath of a child after 24 hours [51] as one of the strategies for newborn thermal care at delivery.

Among other maternal and child related factors, previous birth interval was significantly associated with both under-five and infant mortality. Children who had longer birth intervals had less odds of mortality than those children who had birth interval of less than 24 months. Studies in developing countries also showed inverse association between the length of preceding birth interval and under-five mortality [9, 31, 32, 52]. This may be because of maternal depletion, as women with short intervals between two pregnancies may have insufficient time to restore their nutritional reserves and may suffer maternal depletion, a situation which is thought to adversely affect fetal growth. The latter is expressed in low birth weight or small size for gestational age [30] which in turn have a negative effect on child survival [47]. Competition for resources among the siblings may be another explanation for the association. Therefore, this finding strengthens the importance of adhering to WHO recommendation of at least 24 months birth spacing [30] through promotion of family planning services, specially uptake of post-partum family planning methods.

In this study, history of death of older siblings of the index child was shown to significantly increase the odds of mortality among under-five children and infants. Similarly, higher risk of under-five mortality was reported among children who had death of an older sibling by other studies [52–55]. This may be because of the fact that, factors responsible for the death of previous child sustain and result in the death of the index child. Therefore, reduction of under-five mortality may have twofold advantages as child death is shown to be associated with maternal mental distress[56–58], which in turn is shown to have effect on child wellbeing and survival [59, 60].

Although multiple births are relatively rare events compared to singleton births, they are reported to be a risk factor for under-five mortality, particularly in resource limited settings [52, 61–63]. In the current study, those under-five children, including infants, whose births were multiple had higher rate of odds of mortality than those who were singleton births. So, these findings indicate the importance of meticulous identification and investigation of high risk pregnancies, including multiple pregnancies, during prenatal period in order to take appropriate action.

History of live birth after the index child was examined only for under-five children in order to account for the time required to give birth after birth of the index child. And it was significantly associated with under-five mortality that, under-five children of mothers with history of birth after the index child had higher rate of odds of mortality than those without. This may be because of resource competition for feeding and care. However, birth after index child may be as a consequence of an intention to replace the dead child rather than it being a cause of child mortality. This calls for further investigation with researches that employ stronger study designs.

Finally, even though the two districts for this study were selected purposively, we assumed that the Arba Minch Zuria District and Arba Minch Town are appropriate to represent the population of the Zone. Owing to the study included kebeles from urban-rural, from DSS-non-DSS and from different agro-ecological and climatic conditions; the findings of this study can be generalized to other areas of the country, especially to communities with similar context.

## Conclusions

Among factors classified as ‘personal illness control’, having at least four ANC follow-up visits during pregnancy, utilization of PNC, immunization status of the child and supplementation of Vitamin A for eligible children were significantly associated with under-five mortality. Therefore, these interventions should be strengthened and sustained as strategies of child survival interventions during the SDGs era to maintain reduction of under-five deaths.

Similar to other studies conducted in the country, the current study showed insignificant association between child hood mortality and place and attendants of delivery. So, these findings may trigger a question on the quality of delivery services being provided by health facilities in the study area for further investigation in order to extract the expected positive effects of those health services.

As delaying first bath of a child at least to 24 hours after birth was shown to reduce odds of under-five mortality, its advocacy and strengthening it as one of the strategies of a newborn thermal care at delivery is crucial.

Previous birth interval was among maternal and child factors significantly associated with under-five mortality. Besides, birth after index child was shown to increase odds of mortality among under-five children. So, advocacy of family planning in general and post-partum family planning in particular to delay inter-birth intervals at least to 24 months may be crucial not only for the subsequent birth, but also for the older children.

Children who were from multiple births had higher rate of odds of mortality than those who were singletons. Meticulous identification and investigation of high risk pregnancies, including multiple pregnancies, during prenatal period in order to minimize the negative effect of such risk factors is important.

## Supporting information

S1 Appendix English and Amharic version of the questionnaire. (DOCX)
