# Supplementary material for: Maternal and child characteristics and health practices affecting under-five mortality: A matched case control study in Gamo Gofa Zone, Southern Ethiopia
Source: PLoS One. 2018 Aug 15;13(8):e0202124. doi: 10.1371/journal.pone.0202124 (PMC6093655; doi:10.1371/journal.pone.0202124)
Supplement: S1 Appendix — (DOCX) [file pone.0202124.s001.docx]

### Annex 1: English and Amharic version of the questionnaire

## English version of the questionnaire

District________________ Kebele________________ Gote___________________

House number____________________Child ID_______________Mother ID_______________

Survey questionnaire to determine determinants of childhood mortality and its association with maternal mental distress in Gamo Gofa Zone, Southern Ethiopia

My name is ____________________________. I am working with a research team from Addis Ababa University. We are interviewing systematically selected women/care givers of children about determinants of child mortality and related issues. The purpose of the study is to assess the extent and determinants of child mortality and its association with maternal mental distress, which is expected to be helpful to design, plan and implement programs aiming to reduce child mortality and maternal mental illnesses. I am going to ask you some questions that are very important for the fulfillment of the research. Your answers are completely confidential. Your name or other identifications will never be used in connection with any of the information you tell me. You do not have to answer any question that you do not want to answer, and you may end this interview at any time you want to. However, your honest answers to these questions will help to fulfill the study and to provide information to the programmers to design, plan and implement appropriate interventions. We would greatly appreciate your help in responding to this survey. If you have additional questions/concerns, you can call the principal investigator Girma Temam through 0911959683.

1. Are the information/ objectives clear?

1. Yes 2. No explain again

1. Would you be willing to participate?
2. Yes continue the interview
3. No stop and go to the next HH

Thank you

1. Interviewer’s signature certifying that, the informed consent has been given by the respondent

Name____________________ Signature _____________ Date_______________

1. Result of the questionnaire
2. Completed 2. Refused 3. Partially completed 4. Other__________________
3. Type of participant
4. Child dead 2. Child alive

**Part I: Socio-Economic and Demographic characteristics**

| No | Questions | Coding/ classifications | Skip to | Remark |
| --- | --- | --- | --- | --- |
| 1. 1 | Sex of the respondent | Male……………1  Female…………2 |  |  |
| 1. 1 | Age of the respondent | ­­­­­­­­­­­­­____________________years |  |  |
|  | What is your relationship to the child? | Mother……………………….….........1  Father……………………….…..…….2  Brother/sister………………..….….….3  Grandparent………………….….….…4  Other relative (specify) ____________ 5 | 106 |  |
|  | If the answer to Q103 is not 1, does the mother of the child alive? | Alive……………………………………1  Died ……………………………………2  Don’t know…………………...……….99 | 106  106 |  |
|  | If the mother died, when was she died?  (more than 1 response is possible) | During labor…………………………….1  Immediately after birth……………….…2  With in two months of birth…………….3  After two months of birth………………4  After the child was died………………...5 |  |  |
|  | What is the sex of the child? | Male……………………………………1  Female…………………………………2 |  |  |
|  | When was the child born? | DD/MM/YYYY_____/_____/_________ |  |  |
|  | What was the age of the mother at the time the baby born? | _______________years  Don’t know ……………………… 99 |  |  |
|  | What was the mother’s religion when the child was born? | Protestant ………………………………….1  Orthodox …………………………………..2  Muslim……………………………………..3  Catholic………………………………….....4  Others (specify)______________________5 |  |  |
|  | What is the current religion of the mother? | Protestant ………………………………….1  Orthodox …………………………………..2  Muslim……………………………………..3  Catholic………………………………….....4  Others (specify)______________________5 |  |  |
|  | To which ethnic group the mother belongs to? | Gamo..........................................................1  Gofa............................................................2  Wolayita......................................................3  Zeise ..........................................................4  Amhara .......................................................5  Ganjule........................................................6  Other (specify)…………………………….7 |  |  |
|  | What was the mother’s completed educational status when the child was born? | Illiterate……………………………………1  Read and write …………………………….2  Grade 1 to 6 ……………………………….3  Grade 7 to 8 ……………………………….4  Grade 9 to 12 ………………………………5  Above grade 12…………………………….6 |  |  |
|  | What is the mother’s current completed educational status? | Illiterate……………………………………1  Read and write …………………………….2  Grade 1 to 6 ……………………………….3  Grade 7 to 8 ……………………………….4  Grade 9 to 12 ………………………………5  Above grade 12…………………………….6 |  |  |
|  | What was the mother’s occupation when the child was born? | Farmer……………………………………...1  House wife …………………………….....2  Government employee……………………3  Merchant………………………………….4  Jobless ……………………………………5  Housemaid ……………………………….6  Student ……………………………………7  Others (Specify)______________________8 |  |  |
|  | What is the mother’s current occupation? | Farmer……………………………………...1  House wife …………………………….....2  Government employee……………………3  Merchant………………………………….4  Jobless ……………………………………5  Housemaid ……………………………….6  Student ……………………………………7  Others (Specify)_____________________8 |  |  |
|  | What was the mother’s marital status when the child was born? | Married……………………………………1  Single………………………………………2  Divorced…………………………………..3  Widowed………………………………….4  Separated………………………………….5 | 120  120  120  120 |  |
|  | If married to Q116, how many wives did the husband has including the mother, when the child was born? | The mother only……………………………1  Two…………………...……………………2  Three ………………………………...…….3  Four or more……………………………….4 |  |  |
|  | What was the husband’s occupation when the child was born? | Farmer……………………………………...1  Government employee……………..………2  Merchant……………………………..…….3  Jobless ………………………………..……4  Housemaid …………………………..…….5  Student ……………………………….……6  Others (Specify)______________________7 |  |  |
|  | What was the husband’s educational status when the child was born? | Illiterate……………………………………1  Read and write …………………………….2  Grade 1 to 6 ……………………………….3  Grade 7 to 8 ……………………………….4  Grade 9 to 12 ………………………………5  Above grade 12…………………………….6 |  |  |
|  | What is the mother’s current marital status? | Married……………………………………1  Single………………………………………2  Divorced…………………………………..3  Widowed………………………………….4  Separated………………………………….5 | 124  124  124  124 |  |
|  | If married to Q120, currently how many wives does the husband have including the mother? | The mother only……………………………1  Two…………………...……………………2  Three ………………………………...…….3  Four or more……………………………….4 |  |  |
|  | What is husband’s current occupation? | Farmer……………………………………...1  Government employee……………..………2  Merchant……………………………..…….3  Jobless ………………………………..……4  Housemaid …………………………..…….5  Student ……………………………….……6  Others (Specify)______________________7 |  |  |
|  | What is the husband’s current educational status? | Illiterate……………………………………1  Read and write …………………………….2  Grade 1 to 6 ……………………………….3  Grade 7 to 8 ……………………………….4  Grade 9 to 12 ………………………………5  Above grade 12…………………………….6 |  |  |
|  | What was the main source of income of the family when the child was born? | Labor ………………………………………1  Farming of cash crops……………..………2  Other(specify)_______________________3 |  |  |
|  | What is the main source of income of the family now? | Labor ………………………………………1  Farming of cash crops……………..………2  Other(specify)_______________________3 |  |  |
|  | What was the family’s monthly income, when the child was born? | _____________________Birr per month  No income ……………………………….1  No response………………………………98 |  |  |
|  | What is the family’s monthly income now? | _____________________Birr per month  No income ……………………………….1  No response………………………………98 |  |  |
|  | If you compare the monthly income of the family with the neighbors, where do you put the economic status of the family when the child was born? | Very poor…………………………………..1  Poor………………………….……………..2  Medium…………………………………….3  Rich…………...……………………………4  I can’t say………………….……………….5  No response…………………...…………..98 |  |  |
|  | If you compare the current monthly income of the family with the neighbors, where do you put the economic status of the family? | Very poor…………………………………..1  Poor………………………….……………..2  Medium…………………………………….3  Rich…………...……………………………4  I can’t say………………….……………….5  No response…………………..…………..98 |  |  |

**Part II: Housing condition**

|  | How many family members were living in the house when the child was born? | ________________number |  |  |
| --- | --- | --- | --- | --- |
|  | Currently, how many family members are living in the house? | ________________number |  |  |
|  | What was the roof of the house made of, when the child was born? | Corrugated iron……………………………1  Thatched……………………………………2  Other(specify________________________3 |  |  |
|  | Currently, what is the roof of the house made of? | Corrugated iron……………………………1  Thatched……………………………………2  Other(specify________________________3 |  |  |
|  | Who was the owner of the house when the child was born? | Personal……………………………………1  Rental………………………………………2  Others, specify_______________________3 |  |  |
|  | Currently the owner of the house is? | Personal……………………………………1  Rental………………………………………2  Others, specify_______________________3 |  |  |
|  | How many rooms did the house have, when the child was born? | _______________________number |  |  |
|  | How many rooms does your house have now? | _______________________number |  |  |
|  | Did the house have a window when the child was born? | Yes…………….……………….1  No…………………………..….2 |  |  |
|  | Does the house have a window now? | Yes…………….…………....….1  No……………………………...2 |  |  |
|  | Did animals live with humans in the same housing unit when the child was born? | Yes…………….……………….1  No…………………………..….2 |  |  |
|  | Do animals live with humans in the same housing unit now? | Yes…………….……………….1  No…………………………..….2 |  |  |
|  | Was the cooking usually done in the house, in a separate building, or outdoors when the child was born? | In the house……………………..…1  In a separate building…………..…..2  Outdoors………………………..…...3  Other (specify)______________________4 |  |  |
|  | Did the house have a separate kitchen when the child was born? | Yes…………….……………….1  No…………………………..….2 |  |  |
|  | Is the cooking usually done in the house, in a separate building, or outdoors now? | In the house……………………..…1  In a separate building…………..…..2  Outdoors………………………..…...3  Other (specify)______________________4 |  |  |
|  | Does the house have a separate kitchen now? | Yes…………….……………….1  No…………………………..….2 |  |  |
|  | Did the household have the followings, when the child was born? | YES NO  Electricity? 1 2  A radio? 1 2  A television? 1 2  A mobile telephone? 1 2  A non-mobile telephone? 1 2  A refrigerator 1 2 |  |  |
|  | Does the household currently have:  Electricity?  A radio?  A television?  A mobile telephone?  A non-mobile telephone?  A refrigerator? | YES NO  Electricity? 1 2  A radio? 1 2  A television? 1 2  A mobile telephone? 1 2  A non-mobile telephone? 1 2  A refrigerator 1 2 |  |  |
|  | What was the major source of lighting facility for the house when the child was born? | Electricity………………….…..…………1  Fanos………………………………….….2  Kerosene lamp……………….…………...3  Other (specify)______________________4 |  |  |
|  | What is the major source of lighting facility for the house now? | Electricity………………….……………1  Fanos…………………………………….2  Kerosene lamp……………….…………..3  Other (specify)______________________4 |  |  |
|  | What was the major source of fuel for cooking food in the house, when the child was born? | Wood ……………………………………..1  Anima dung……………………………….2  Charcoal…………………………………..3  Kerosene…………………………………..4  Electricity………………………………….5  Others, specify_______________________6 |  |  |
|  | What is the major source of fuel for cooking food in your house now? | Wood ……………………………………..1  Anima dung……………………………….2  Charcoal…………………………………..3  Kerosene…………………………………..4  Electricity………………………………….5  Others, specify______________________6 |  |  |
|  | Did any member of your household own any of the followings when the child was born? | Yes/No if yes, number  Bicycle _______ ________  Motorcycle ________ ________  Car _______ ________  Livestock _______ ________  Pack animals _______ ________  Sheep/goats _______ ________  Chicken (poultry) _______ ________ |  |  |
|  | Does any member of your household currently own: Bicycle?  Motorcycle?  Car?  Livestock?  Pack animals?  Sheep/goats?  Chicken (poultry)? | Yes/No if yes, number  Bicycle _______ ________  Motorcycle ________ ________  Car _______ ________  Livestock _______ ________  Pack animals _______ ________  Sheep/goats _______ ________  Chicken (poultry) _______ ________ |  |  |

**Part III: Water and sanitation**

|  | What was the source of drinking water for your family when the child was born? | Tap………………………………………...1  Protected well/ spring…………………..…2  Unprotected well/spring ……………….…3  River/pond…………………………….…..4  Other (specify)_______________________5 |  |  |
| --- | --- | --- | --- | --- |
|  | Did you do anything to the water to make it safer to drink, when the child was born? | Yes…………….…………………..…….1  No…………………………………….….2 | 304 |  |
|  | If yes to Q302, how? | Boiling……………………………….……1  Chemicals…………………………………2  Sand filter………………………………….3  Others, specify______________________4 |  |  |
|  | Current source of drinking water of the family is? | Tap……………………………………..….1  Protected well/ spring…………………..…2  Unprotected well/spring ……………….…3  River/pond………………………………...4  Other (specify)_______________________5 |  |  |
|  | Do you do anything to the water to make it safer to drink now? | Yes…………….…………………..…….1  No…………………………………….….2 | 307 |  |
|  | If yes to Q305, how? | Boiling ……………………………………1  Chemicals…………………………………2  Sand filter…………………………………3  Others, specify______________________4 |  |  |
|  | Did the family have latrine facility when the child was born? | Yes…………….…………………….….1  No……………………………………….2 | 310 |  |
|  | If yes, What was the type of the latrine? | Simple Pit………………………………1  VIP……………………………………..2  Flush toilet……………………………..3  Other (specify)_______________________4 |  |  |
|  | Was the latrine shared with other households or not? | Shared……………………………………..1  Not shared…………………………………2  Other (specify)_______________________3 |  |  |
|  | Does the family have latrine facility now? | Yes…………….………………..……….1  No……………………………………….2 | 401 |  |
|  | If yes, What is the type of the latrine? | Simple Pit…………………….…………1  VIP………………………….…………..2  Flush toilet…………………….………..3  Other (specify)_______________________4 |  |  |
|  | Is the latrine shared with other households or not | Shared……………………………………..1  Not shared…………………………………2  Other (specify)_______________________3 |  |  |
|  | Cleanliness of the latrine (Observe) | Clean………………………………………1  Not clean ………………………………….2 |  |  |

**Part IV: Reproductive History of the mother**

|  | Had ever the mother been pregnant prior to the pregnancy of the index child? | Yes…………….…………………..…….1  No………………………………………..2 | 413 |  |
| --- | --- | --- | --- | --- |
|  | If yes, how many pregnancies the mother had before the index child? | __________________Number |  |  |
|  | How many of these pregnancies ended up with:  Abortion?  Still birth?  Live birth? | Number  Abortion____________________  Still birth_____________________  Live birth____________________  Don’t know____________________ |  |  |
|  | What was the outcome of the pregnancy just prior to the index child? | Abortion………………………..….1  Still birth……………………….…..2  Live birth……………………….….3  Don’t know……………………….99 |  |  |
|  | What is the interval between the termination of the preceding pregnancy and the birth of index child? | ____________________months |  |  |
|  | What is the interval between the termination of the preceding pregnancy and the pregnancy of the index child? | ____________________months |  |  |
|  | If not live birth to Q404, What is the interval between the preceding live birth and the index birth? | ____________________months |  |  |
|  | Did there any child who is older than this child died? | Yes…………….……………………...1  No………………………………….….2 | 411 |  |
|  | If yes to Q409. How many died? | _______________________number |  |  |
|  | What is the birth order of the index child? | First .……………………….……………1  Second ……………………………….….2  Third ………………………………........3  Fourth or higher …………………………4  Don’t know ………………………….....99 | 413 |  |
|  | If the birth order of the child is more than one, how many alive children were the mother has when the child was born? | _______________________number |  |  |
|  | Does any child born after the index child? | Yes…………….……………………..….1  No……………………………………….2 | 418 |  |
|  | If yes to Q413. How many? | _______________________number |  |  |
|  | After how many months of the birth of the index child, the child next to the index child born? | ______________months |  |  |
|  | Is there any child who is younger than this child died? | Yes…………….……………………….1  No………………………………..…….2 | 418 |  |
|  | If yes to Q416. How many died? | _______________________number |  |  |
|  | How many children does the mother have now? | _______________________number |  |  |
|  | Are you pregnant now? | Yes …………………….………………... 1  No …………………………………….… 2  Unsure…………………………………….3 | 501  501 |  |
|  | How many months pregnant are you now? | __________________months |  |  |

**Part V: Maternal and child health service utilization**

|  | Did the mother receive antenatal care for the pregnancy of the index child? | Yes ……………………………….……...1  No ………………………………….…….2  Don’t know ……………………………..99 | 506  506 |  |
| --- | --- | --- | --- | --- |
|  | Where was the ANC service obtained? | Hospital…………………………….….....1  Health Center……………………………2  Health Post…………………..….……….3  Other (specify)….. …... ………………..4  Don’t know ……………………………..99 |  |  |
|  | When was the first ANC checkup made during the pregnancy? | Within the first 3 months…………..…….1  Within the 1^st^ 6 months…………………..2  After 6^th^ months of pregnancy……….…..3  Don’t know……………………………..99 |  |  |
|  | How many times the mother received antenatal care during the index pregnancy? | ____________________Number  Don’t know…………………………….99 |  |  |
|  | Who provided the ANC service? | Doctor /HO…………………………..…....1  Nurse/midwife……………………….…....2  HEW…………………………………....…3  Trained TBA…………………………...….4  Traditional birth attendant…………..….... 5  Other (specify)_______________________9  Don’t know …………………..………….99 |  |  |
|  | Did the mother received tetanus toxoid (TT) vaccine? | Yes …………………………………....1  No ………………………………….….2  Don’t know…………………………...99 | 508  508 |  |
|  | Which dose of TT she had taken?  (from card or history) | TT1………………………………….….....1  TT2………………………………….…....2  TT3………………………………….…....3  TT4………………………………….…....4  TT5………………………………….…....5  Don’t know …………………………… 99 |  |  |
|  | Where was the child born? | Home ………………………………….…....1  Hospital……………………………….….…2  Health Center………………………………3  Health Post…………………..……….….…4  Other health facility …... ……………..…..5  Other (specify)________________________7 Don’t know ………………………..…......99 |  |  |
|  | Who assisted with the delivery? | Health worker……………………………. 1  Health Extension Worker ……………….. 2  Trained TBA…………………………..….3  Traditional birth attendant ………………. 4  Relative………………………………...…..5  Neighbor……………………………..……6  Mother by herself ………….………...…..7  Other_______________________________8  Don’t know……………………………….99 |  |  |
|  | Was the child a single or multiple birth? | Singleton……………………..…….….1  Twin …………………………………..2  Triplet or more ………………………..3  Don’t know …………………………...99 |  |  |
|  | When the child was born, was he/she very large, larger than average, average, smaller than average, or very small? | very large……………………………….1  larger than average………………..……2  average………………………..…….….3  smaller than average…………..……….4  very small………………………..……..5  Don’t know …………………………...99 |  |  |
|  | Did the mother’s and/or the child’s health checked by health professional after birth of the index child? | Yes…………………………………..…..1  No………………………………….…….2 | 517 |  |
|  | If yes, who checked her own and/or her child’s health status? | Health worker……………………………. 1  Health Extension Worker ……………….. 2  Trained TBA…………………………..….3  Traditional birth attendant ………………. 4  Relative………………………………...…..5  Neighbor……………………………..……6  Mother by herself ………….………...…..7  Other_______________________________8  Don’t know……………………………….99 |  |  |
|  | Where was the checkup made? | Hospital……………………………….....1  Health Center……………………..….…2  Health Post…………………..………….3  Home…………………..…………..……4  Other (specify______________________5  Don’t know …………………………..99 |  |  |
|  | How long after delivery did the first check take place? | ____________________Hours  ____________________days  ____________________weeks |  |  |
|  | How many times such checkup was made before 42 weeks of birth? | Once……………………………………..1  At two occasions…………………..…….2  At three occasions…………………….…3  More than three occasions………………..4  Don’t know ……………………………..99 |  |  |
|  | Did the index child get vaccination | Yes……………………………………..1  No………………………………..…….2 | 520 |  |
|  | If yes to Q517, which vaccines the child has taken so far? (from card or history) | Yes No  Polio0 ______ ______  BCG ______ ______  Polio1 ______ ______  Polio2 ______ ______  Polio3 ______ ______  Penta1 ______ ______  Penta2 ______ ______  Penta3 ______ ______  Measles ______ ______ |  |  |
|  | If yes to Q517, what is/was the immunization status of the child? (from card or history) | Fully vaccinated……………..…………1  Partially vaccinated…………….………2  Not vaccinated at all……………………3  Don’t know…………………………….99 |  |  |
|  | Did the index child receive a vitamin A like this? (from card or history) | Yes…………………………………..…..1  No……………………………………….2  Don’t know…………………………….99 | 522  522 |  |
|  | If yes to Q520. How many times? | _______________________number |  |  |
|  | Please tell me at which of the following places or facilities usually the family seek treatment. | YES NO DK  Home …………………….. 1 2 8 Traditional healer……... ….... 1 2 8 Government Health Post.……...1 2 8  Government Health center….....1 2 8  Government hospital ……...…1 2 8 Private clinic …..……………..1 2 8 Private hospital……………… 1 2 8  Pharmacy, drug seller, store….1 2 8  Other(Specify)_______________________ |  |  |
|  | Did the mother have history of chronic medical illness during the pregnancy of the index child? | Yes………………………………………..1  No……………………………………..….2 | 525 |  |
|  | If yes, what were the problems?  (multiple responses are possible) | Diabetes mellitus …………………..…....1  Cardiac Disease………………………… 2  Hypertension ……………………………..3  Renal Disease …………………………….4  Other( Specify) _____________________5 |  |  |

**Part VI. Child feeding and new born care practice**

|  | Was the child ever breastfeed? | Yes……………………………………...1  No…………………………………….....2 | 604 |  |
| --- | --- | --- | --- | --- |
|  | How long after birth did the child put to the breast? | _________hrs  _________days |  |  |
|  | How long did the child breast feed? | Still breast feeding  __________months |  |  |
|  | In the first three days after delivery, was the child given anything other than breast milk? | Yes………………………………………..1  No……………………………………..….2  No response……………..……………….98 | 606  606 |  |
|  | If yes to Q604, what was given? | Milk (other than breast milk) . . …..…….1  Plain water . . . . . . . . …………………....2  Sugar-salt-water solution . . . . . …………3  Fruit juice . . . . . . . . ………………..……4  Infant formula . . . . ……………….……..5  Honey . . . . . . . . . . . . …………….……..6  Fresh butter . . . . . . ………………………7  Other (specify)______________________8 |  |  |
|  | In the first 6months after delivery, was the child given anything to drink and/or eat other than breast milk? | Yes………………………………………..1  No……………………………………..….2  No response……………..……………….98 | 609  609 |  |
|  | What was given to drink and/ or eat in the first 6 months? | Plain water . . . . . . . . …………….………..1  Milk (other than breast milk) . . . ….….. ….2  Gruel………………… . . . . . ………...……3  Fresh butter . . . . . . …………………..……4  Sugar-salt-water solution . . . ... ……………5  Fruit juice . . . . . . . . …………………….…6  Infant formula . . . . ………………………..7  Tea/infusions . . . . . . ……….……..………8  Honey . . . . . . . . . . . . ……………………..9  Adult foods………………………………10  Other (specify)______________________11 |  |  |
|  | After how long after birth, it was started? | _____________days  ____________months |  |  |
|  | Did the child drink anything from a bottle with a nipple? | Yes…………………………..………..…..1  No………………………………….…..….2  No response……………………...……….98 |  |  |
|  | If the child is older than 6months, did the child have been given additional food other than breast milk? | Yes…………………………..…………..1  No………………………………….…….2  No response……………………...……….98 | 613  613 |  |
|  | When was additional food started after birth? | ___________________days  _________________months |  |  |
|  | What was the additional food give to the child | Cow milk…………………………………1  Gruel……………………………….……..2  Adult foods………………………….……3  Other(specify)_______________________4 |  |  |
|  | When was the child given first bath after birth? | Immediately after delivery………………..1  After an hour of delivery………………….2  After 6 hours………………………………3  After 12 hours……………………………4  After 24 hours……………………………5  After 48 hours……………………………6 |  |  |
|  | Was there anything applied at the umbilical wound of the child after birth? | Yes………………………………………..1  No……………………………………..….2  No response……………..……………….98 | 616  116 |  |
|  | If yes to Q614, what was applied? | Butter…………………………………....…1  Animal dung……………………….…….…2  Other (specify)________________________3 |  |  |
|  | Did the child ever get sick? | Yes………………………………………..1  No……………………………………..….2  No response……………..……………….98 | 619  619 |  |
|  | If yes, did s/he have any treatment? | Yes………………………………………..1  No……………………………………..….2  No response……………..……………….98 | 619  619 |  |
|  | Where did s/he get the treatment | Home……………………………….…….1  Traditional healer…………………………2  Health Post ………………………….……3  Health Center…………………….……….4  Hospital………………………….………..5  Private clinic…………….…….…………..6  Private pharmacy………………………….7  Others(specify)……………………………8 |  |  |
|  | Were the followings done for the child after birth? | Yes No  Uvulectomy 1 2  Milk teeth extraction 1 2  Skin piercing/burning 1 2  Other(specify)________________________ |  |  |

**Part VII: Woman’s status**

|  | Did the mother earn any sort of cash either by working or selling house goods, when the child was born? | Yes………………………………………..1  No……………………………………..….2  No response……………..……………….98 | 703  703 |  |
| --- | --- | --- | --- | --- |
|  | Who usually did decide how the money she earns will be used when the child was born? | The mother…………………………………1  Her husband/partner……………………….2  She and her husband/partner jointly………3  Other (specify)_______________________4 |  |  |
|  | Does the mother earn any sort of cash either by working or selling house goods now? | Yes………………………………………..1  No……………………………………..….2  No response……………..……………….98 | 705  705 |  |
|  | Who usually decides how the money she earns will be used now? | The mother…………………………………1  Her husband/partner……………………….2  She and her husband/partner jointly………3  Other (specify)_______________________4 |  |  |
|  | Who usually did decide how the husband’s earnings will be used when the child was born? | The mother…………………………..…….1  Her husband/partner………………...……..2  She and her husband/partner jointly……….3  The husband has no earnings………………4  Other (specify)_______________________5 |  |  |
|  | Who usually decides how the husband’s earnings will be used now? | The mother…………………………………1  Her husband/partner……………………….2  She and her husband/partner jointly………3  The husband has no earnings………………4  Other (specify)_______________________5 |  |  |
|  | Who usually made decisions about health care for the mother when the child was born? | The mother………………..……………….1  Her husband/partner……………………….2  She and her husband/partner jointly………3  Someone else………………………………4  Other (specify)_______________________5 |  |  |
|  | Who usually makes decisions about health care for the mother now? | The mother………………..……………….1  Her husband/partner……………………….2  She and her husband/partner jointly………3  Someone else………………………………4  Other (specify)_______________________5 |  |  |
|  | Who usually made decisions about making major household purchases when the child was born? | The mother………………..……………….1  Her husband/partner……………………….2  She and her husband/partner jointly………3  Someone else………………………………4  Other (specify)_______________________5 |  |  |
|  | Who usually makes decisions about making major household purchases now? | The mother………………..……………….1  Her husband/partner……………………….2  She and her husband/partner jointly………3  Someone else………………………………4  Other (specify)_______________________5 |  |  |
|  | Who usually made decisions about visits to her family or relatives when the child was born? | The mother………………..……………….1  Her husband/partner……………………….2  She and her husband/partner jointly………3  Someone else………………………………4  Other (specify)_______________________5 |  |  |
|  | Who usually makes decisions about visits to her family or relatives now? | The mother………………..……………….1  Her husband/partner……………………….2  She and her husband/partner jointly………3  Someone else………………………………4  Other (specify)_______________________5 |  |  |
|  | Did the husband help the mother with household chores like looking after the children, cooking, cleaning the house, and doing other work around the house when the child was born? | Yes………………………………….……..1  No………………………………………….2  No response……………………….……….98 |  |  |
|  | Does the husband help the mother with household chores like looking after the children, cooking, cleaning the house, and doing other work around the house now? | Yes………………………………….……..1  No………………………………………….2  No response……………………….……….98 |  |  |
|  | In your opinion, is a husband justified in hitting or beating his wife in the following situations:  If she goes out without telling him?  If she neglects the children?  If she argues with him?  If she refuses to have sex with him?  If she burns the food? | Yes No DK  Goes out . . . . . . . . . .…..1 2 8  Negl. Children. . ……… 1 2 8  argues . . . . . . . . . . . .…..1 2 8  Refuses sex . . . . . …….. 1 2 8  Burns food . . . . . . . .….. 1 2 8 |  |  |
|  | Was your opinion different from the above (Q715), when the child was born? | Yes……………………….…….…………..1  No………………………………………….2 | 718 |  |
|  | If yes to Q716, in which acts? | Yes No DK  Goes out . . . . . . . . . .…..1 2 8  Negl. Children. . ……… 1 2 8  Argues . . . . . . . . . . . …..1 2 8  Refuses sex . . . . . …….. 1 2 8  Burns food . . . . . . . .….. 1 2 8 |  |  |
|  | Had the mother ever been beaten by her husband when the child was born? | Yes………………………………….……..1  No………………………………………….2  No response……………………….……….98 |  |  |
|  | Has the mother ever been beaten by her husband now? | Yes………………………………….……..1  No………………………………………….2  No response……………………….……….98 |  |  |
|  | Is there a law in Ethiopia that prevents a husband from beating his wife? | Yes……………………………….……..…..1  No………………………………………..….2  Don’t know….……………………….…….99 |  |  |

I have finished my questions.

Thank you very much.

**Checked by supervisor:** Name_______________ Signature ___________ Date______________

## Amharic version of the questionnaire

ወረዳ______________________ ቀበሌ____________________ ጎጥ___________________

የቤት ቁጥር________________________

የፈቃደኝነት መጠየቂያና ማረጋገጫ ፎርም

ስሜ____________________________ይባላል፡፡ በአዲስ አበባ ዩኒቨርስቲ የጥናት ቡድን አባል ነኝ፡፡ በተመሳሳይ ሁኔታ ለተመረጡ እናቶች ስለ ልጆች ሞት መንስኤ ስለሆኑና ሌሎች ተዛመጅ ጉዳዮች ቃለ መጠይቅ እያደረግን ነው፡፡ የጥናቱ አላማ ለልጆች ሞት መንስኤ የሆኑ ጉዳዮችና የእናቶች የአይምሮ ጤንነት ሁኔታና ተያያዥነት ያላቸው ጉዳዮችን ለይቶ ለማውጣት ሲሆን ጥናቱ የልጆችን ሞት ለመቀነስ በመንግስትም ሆነ መንግስታዊ ባልሆኑ ድርጅቶች ለሚሰሩ ስራዎች ግባት በመሆን የልጆችን ሞት ለመቀነስና የናቶችን የአይምሮ ጤንነት በመጠበቅ ረገድ ከፍተኛ አስተዋጽኦ ይኖረዋል፡፡ አሁን ለጥናቱ የሚያስፈልጉ የተወሰኑ ጥያቄዎችን አቀርብሎታለሁ፡፡ መልስዎ ምስጢራዊነቱ ከማንኛውም አካል የተጠበቀ ነው፡፡ ስምዎም ሆነ ሌሎች የእረስዎ መለያ በዚህ ፎርም ላይ አይሞላም ወይንም አይጻፍም፡፡ ከሌላ ከሚነግሩኝ መረጃ ጋርም አይያያዝም፡፡ መመለስ የማፈልጉትን ጥያቄ የግድ መመለስ የለብዎትምና መተው ይችላሉ፡፡ እንዲሁም ካልፈለጉ በማንኛውም ሰዓት ቃለ መጠይቁን ማቋረጥ ይችላሉ፡፡ በቓለ መጠይቁ በመሳተፍዎ የሚያገኙት የተለየ ክፍያ የለም፡፡ እንዲሁም ባለ መሳተፍዎ የሚደርስብዎት ምንም አይነት ችግር የለም፡፡ ነገር ግን ለጥያቄዎቹ እርስዎ የሚሰጡንን ቅንና ትክክለኛ መልስ ጥናቱ የተሟላ እንዲሆን ከማድረጉም በላይ ለውሳኔ ሰጭዎችና ለጤና ባለሙያዎች አስፈላጊውን መረጃ በመስጠት የህጻናትን ሞትና ህመም ለመቀነስ እንዲሁም የእናቶችን የአይምሮ ጤና ለማጎልበት በሚደረገው እንቅስቃሴ ከፍተኛ ጠቀሜታ ይኖረዋል፡፡ ለጥቄዎቹ ለሚሰጡን ምላሽ አድናቆታችን በጣም ከፍ ያለ ነው፡፡

ተጨማሪ ጥያቄ ካለዎት የጥናቱ ዋና አስተባባሪ የሆኑትን ግርማ ተማም በ 0911959683 በመደወል ማነጋገር ይችላሉ፡፡

ሀ） ግልጽ ነው？ 1. አዎ 2. አይደለም በድጋሚ ያብራሩላቸው

ለ） በጥናቱ ለመሳተፍ ፍቃደኛ ነዎት ?

1. አዎ መጠይቁን ይቀጥሉ

2. አይደለሁም መጠይቁን ያቁሙና ወደ ቀጣዩ ቤት ይሂዱ :

ሐ） የቃለ መጠይቅ አድራጊው ስምና ፊርማ

ስም----------------------------------------------ፊርማ--------------------ቀን-----------------

መ） የቃለ መጠይቁ ውጤት

1） ሙሉ ለሙሉ የተጠናቀቀ 2） ፍቃደኛ አይደሉም 3） በከፊል የተጠናቀቀ 4） ሌላ----------------

የተሳታፊዋ ሁኔታ

ሀ. ልጅ የሞተባት ለ. ልጅ ያልሞተባት

**ክፍል 1፡ ማህበራዊና ኢኮኖሚያዊ ሁኔታዎች**

| ተ ቁ | ጥያቄዎች | የመልስ ምረጫዎች | ዝለል | ምርመራ |
| --- | --- | --- | --- | --- |
|  | የመላሽ ፆታ | ወንድ………………………………………1  ሴት…………………………………..……2 |  |  |
|  | የመላሽ ዕድሜ | ­­­­­­­­­­­­­_______________________ዓመት |  |  |
|  | ከልጁ ጋር ያለዎት ዝምድና | እናት……………………….…………........1  አባት……………………….………...…….2  ወንድም/እህት………………..……….….….3  አያት………………….….……………...…4  ሌላ ዘመድ (ይገለጽ) _______________________ 5 | 106 |  |
|  | ለጥ103 መልስ 1 ካልሆነ፤ የልጁ እናት በህይወት አለች? | አዎ…………………………………..…………1  አይ ሞታለች……………..………………………2  አላውቅም……………………………...……….99 | 106  106 |  |
|  | እናቱ ሞታ ከሆነ፤ መቼ ነበር የሞተችው? | በምጥ ወቅት…………………………………….1  ወዲያው ልጁ ከተወለደ በኋላ………………..….…2  ልጁ በተወለደ በ2 ወር ውስጥ…………………..….3  ልጁ ከተወለደ ከ2 ወር በኋላ………………………4  ልጁ ከሞተ በኋላ………………………………...5 |  |  |
|  | የልጁ ፆታ? | ወንድ…………………………………..……1  ሴት…………………………………………2 |  |  |
|  | ልጁ መቼ ነበር የተወለደው? | ቀን/ወር/አመት_____/_____/_________ |  |  |
|  | ልጁ ሲወለድ እናትየዋ እድሜዋ ስንት ነበር? | _______________አመት  አላውቅም…………………...……….99 |  |  |
|  | ልጁ ሲወለድ የእናትየዋ ሀይማኖት ምን ነበር? | ፕሮሰቴታንት …………………………………..1  ኦርቶዶክስ ……………………………..….…...2  ሙስሊም…………………………….…….…..3  ካቶሊክ…………………………………….......4  ሌላ (ይገለጽ)____________________________5 |  |  |
|  | አሁን የናትየዋ ሃይማኖት ምንድን ነው? | ፕሮሰቴታንት ………………………………….1  ኦርቶዶክስ ………………………………..…..2  ሙስሊም……………………………………..3  ካቶሊክ…………………………………….....4  ሌላ (ይገለጽ)___________________________5 |  |  |
|  | የናትየዋ ብሄር ምንድን ነው? | ጋሞ................................................................1  ጎፋ.................................................................2  ወለይታ...........................................................3  ዘይሴ ............................................................4  አማራ ............................................................5  ጋንጁሌ............................................................6  ሌላ (ይገለጽ)………………………………….7 |  |  |
|  | ልጁ ሲወለድ የእናትየዋ የትምህርት ደረጃ ስንት ነበር? | ያልተማረች……………………………………1  መጻፍና ማንበብ ……………………………….2  1 to 6 ክፍል…………………………….…….3  7 to 8 ክፍል ………………………………….4  9 to 12 ክፍል …………………………………5  ከ12ኛ ክፍል በላይ……………………………….6 |  |  |
|  | አሁን የናትየዋ የትምህርት ደረጃ ስነት ነው? | ያልተማረች……………………………………1  መጻፍና ማንበብ ……………………………….2  1 to 6 ክፍል………………………….……….3  7 to 8 ክፍል ………………………………….4  9 to 12 ክፍል …………………………...……5  ከ12ኛ ክፍል በላይ………………………..…….6 |  |  |
|  | ልጁ ሲወለድ የእናትየዋ የስራ ሁኔታ ምን ነበር? | ገበሬ………………………………………...1  የቤት እመቤት ……………………….…….....2  የመንግስት ሰራተኛ…………………….………3  ነጋዴ……………………………….……….4  ስራ አጥ ………………………….…………5  የሰው ቤት ሰራተኛ…………………………….6  ተማሪ ………………………………………7  ሌላ (ይገለጽ)__________________________8 |  |  |
|  | አሁን የስራዋ ሁኔታ ምንድን ነው? | ገበሬ………………………………………...1  የቤት እመቤት ………………………….….....2  የመንግስት ሰራተኛ………………………….…3  ነጋዴ………………………………..……….4  ስራ አጥ ………………………………..……5  የሰው ቤት ሰራተኛ……………………….…….6  ተማሪ ………………………………….……7  ሌላ (ይገለጽ)__________________________8 |  |  |
|  | ልጁ ሲወለድ የእናትየዋ ትዳር ሁኔታ እንዴት ነበር? | ባለትዳር……………………………………...…1  ያላገባች………………………………….…...…2  የፈታች…………………………………….…..3  ባል የሞተባት………………………………..….4  የተሌች……………………………………..….5 | 120  120  120  120 |  |
|  | ለጥ116 መልስ1 ከሆነ፤ ባልዋ ስንት ሚስት ነበረው? | እሷ ብቻ………………………………………1  ሁለት…………………...………….…………2  ሶስት …………………………………...…….3  አራትና ከዚያ በላይ…………………….………..4 |  |  |
|  | ልጁ ሲወለድ የባልዮ የስራ ሁኔታ ምን ነበር? | ገበሬ…………………………………………...1  የመንግስት ተቀጣሪ……………..……………...…2  ነጋዴ……………………………………..…….3  ስራ አጥ ………………………………..………4  የሰው ቤት ሰራተኛ……………………………….5  ተማሪ …………………………………….……6  ሌላ (ይገለጽ)____________________________7 |  |  |
|  | ልጁ ሲወለድ የባልዮ የትምህርት ደረጃ ምን ነበር? | ያልተማረ…………………………………..…1  መጻፍና ማንበብ ……………………………….2  1 to 6 ክፍል………………………………….3  7 to 8 ክፍል ……………………………...….4  9 to 12 ክፍል ……………………………..…5  ከ12ኛ ክፍል በላይ………………………….….6 |  |  |
|  | አሁን የናትዮዋ የጋብቻ ሁኔታ? | ባለትዳር………………………………….………1  ያላገባች………………………………………..…2  የፈታች…………………………………….……..3  ባል የሞተባት……………………………..…….4  የተለያየች……………………………….…….….5 | 124  124  124  124 |  |
|  | ለጥ121 መልስ1 ከሆነ፤ ባልዋ ስንት ሚስት አለው? | እሷ ብቻ………………………………………1  ሁለት…………………...……………….……2  ሶስት ………………………………...……….3  አራትና ከዚያ በላይ…………………………..….4 |  |  |
|  | አሁን የባልየው የስራ ሁኔታ ምንድን ነው? | ገበሬ………………………………………….....1  የመንግስት ተቀጣሪ…………..……………….…2  ነጋዴ……………………………..…………..….3  ስራ አጥ …………………………………………4  የሰው ቤት ሰራተኛ………………………..……….5  ተማሪ ……………………………….…..………6  ሌላ (ይገለጽ)____________________________7 |  |  |
|  | አሁን የባልየው የትምህርት ደረጃ ምንድን ነው? | ያልተማረ………………………………….…1  መጻፍና ማንበብ …………………….……..….2  1 to 6 ክፍል………………………….…..….3  7 to 8 ክፍል ………………………….….….4  9 to 12 ክፍል …………………………….…5  ከ12ኛ ክፍል በላይ………………………….….6 |  |  |
|  | ልጁ ሲወለድ የቤተሰቡ ዋነኛ የገቢ ምንጭ ምን ነበር? | የጉልበት ስራ…………………………………1  ግብርና……………..………………….……2  ሌላ (ይገለጽ)_________________________3 |  |  |
|  | አሁን የቤተሰቡ ዋነኛ የገቢ ምንጭ ምነድን ነው? | የጉልበት ስራ…………………………………1  ግብርና……………..………………….……2  ሌላ (ይገለጽ)_________________________3 |  |  |
|  | ልጁ ሲወለድ የቤተሰቡ ወርሀዊ ገቢ ስንት ነበር? | _____________________ብር በወር  ገቢ አልነበረውም……………………………….1  ለመመለስ ፍቀደኛ አይደሉም……………………98 |  |  |
|  | አሁን የቤተሰቡ ወርሃዊ ገቢ ስንት ነው? | _____________________ብር በወር  ገቢ የለውም………………………………….1  ለመመለስ ፍቀደኛ አይደሉም……………………98 |  |  |
|  | ልጁ ሲወለድ፤ የቤተሰቡ የገቢ ሁኔታ ከጎረቤች ጋር ሲነፃፀር? | በጣም ድሀ……………………………………..1  ድሀ………………………….…………….…..2  መካከለኛ……………………………………….3  ሃብታም………...………………………………4  መገመት አልችልም……………….……………….5  ለመመለስ ፍቀደኛ አይደሉም……...……………..98 |  |  |
|  | አሁን የቤተሰቡ የገቢ ሁኔታ ከጎረቤት ጋር ሲነፃፀር? | በጣም ድሀ………………………………….…..1  ድሀ………………………….…………….…..2  መካከለኛ…………………………………….….3  ሃብታም………...………………………………4  መገመት አልችልም……………….……………….5  ለመመለስ ፍቀደኛ አይደሉም……...……………..98 |  |  |

**ክፍል2፡ የቤተሰቡ መኖሪያ ቤት ሁኔታ**

|  | ልጁ ሲወለድ በቤቱ ውስጥ ስንት ሰው ይኖር ነበር? | ________________በቁጥር |  |  |
| --- | --- | --- | --- | --- |
|  | አሁን በቤቱ ውስጥ ስንት ሰው ይኖራል? | ________________በቁጥር |  |  |
|  | ልጁ ሲወለድ የቤቱ ጣሪያ የተሰራው ከምን ነበር? | ቆርቆሮ……………………………..…….…1  ሳር………………………………..…..……2  ሌላ (ይገለጽ)_________________________3 |  |  |
|  | አሁን የቤቱ ጣሪያ የተሰራው ከምንድን ነው? | ቆርቆሮ………………………………….……1  ሳር………………………………..…………2  ሌላ (ይገለጽ)__________________________3 |  |  |
|  | ልጁ ሲወለድ የቤቱ ሁኔታ ምን ነበር? | የግል…………………………………………1  የኪራይ………………………………………2  ሌላ (ይገለጽ)_________________________3 |  |  |
|  | አሁን የቤቱ ሁኔታ እንዴት ነው? | የግል…………………………………………1  የኪራይ………………………………………2  ሌላ (ይገለጽ)__________________________3 |  |  |
|  | ልጁ ሲወለድ ቤቱ ስንት ክፍል ነበረው? | _______________________በቁጥር |  |  |
|  | አሁን ቤቱ ስንት ክፍል አለው? | _______________________በቁጥር |  |  |
|  | ልጁ ሲወለድ ቤቱ መስኮት ነበረው? | አዎ…………….………………….1  የለም…………………………...….2 |  |  |
|  | አሁን ቤቱ መስኮት አለው? | አዎ…………….………….……..….1  የለም……………………………..….2 |  |  |
|  | ልጁ ሲወለድ፤ ከብቶች ከሰው ጋረ በአንድ ቤት ውስጥ ይኖሩ ነበር? | አዎ…………….……………….….1  የለም…………………………....….2 |  |  |
|  | አሁን ከብቶች ከሰው ጋረ በአንድ ቤት ውስጥ ይኖራሉ? | አዎ…………….……………..…….1  የለም…………………………….….2 |  |  |
|  | ልጁ ሲወለድ፤ ቤተሰቡ ምግብ ለማብሰል ይጠቀም የነበረው መኖሪያ ቤት ውስጥ፣ በሌላ ክፍል/ቤት ወይም ውጭ ላይ ነበር? | መኖሪያ ቤት ውስጥ…………….…………..…….1  በሌላ ክፍል…………………………………….….2  ከቤት ውጭ………………………………….….3  ሌላ (ይገለጽ)_____________________________4 |  |  |
|  | ልጁ ሲወለድ፤ ቤቱ የተለየ ማድ ቤት/ኩሽና ነበረው? | አዎ…………….………………….1  የለም…………………………...….2 |  |  |
|  | አሁን ቤተሰቡ ምግብ ለማብሰል የሚጠቀመው መኖሪያ ቤት ውስጥ፣ በሌላ ክፍል/ቤት ወይም ውጭ ላይ ነው? | መኖሪያ ቤት ውስጥ…………….…………..…….1  በሌላ ክፍል…………………………………….….2  ከቤት ውጭ………………………………….….3  ሌላ (ይገለጽ)_____________________________4 |  |  |
|  | አሁን ቤቱ የተለየ ማድ ቤት/ኩሽና አለው? | አዎ…………….……………….….1  የለም……………………………….2 |  |  |
|  | ልጁ ሲወለድ፡ ቤተሰቡ ከሚከተሉት የትኞቹ ነበሩት? | አዎ የለም  ኤሌክትሪክ? 1 2  ሬዲዮ? 1 2  ቴሌቬዥን? 1 2  ሞቢል ስልክ? 1 2  የመስመር ስልክ? 1 2  ፍሪጅ? 1 2 |  |  |
|  | አሁን ቤተሰቡ ከሚከተሉት የትኞቹ አሉት? | አዎ የለም  ኤሌክትሪክ? 1 2  ሬዲዮ? 1 2  ቴሌቬዥን? 1 2  ሞቢል ስልክ? 1 2  የመስመር ስልክ? 1 2  ፍሪጅ? 1 2 |  |  |
|  | ልጁ ሲወለድ፡ የቤተሰቡ ዋነኛ የማብራት/የብርሃን ምንጭ ምን ነበር? | ኤሌክትሪክሲቲ………………….……………1  ፋኖስ……………………………………….2  ኩራዝ……………….………………….…..3  ሌላ (ይገለጽ)_________________________4 |  |  |
|  | አሁን፤ የቤተሰቡ ዋነኛ የማብራት/የብርሃን ምነጭ ምንድን ነው? | አሌክትሪክሲቲ………………….……………1  ፋኖስ……………………………………….2  ኩራዝ……………….……………….……..3  ሌላ (ይገለጽ)________________________4 |  |  |
|  | ልጁ ሲወለድ፤ ቤተሰቡ ምግብ ለማብሰል በዋነኛነት ይጠቀም የነበረው ምን ነበር? | እንጨት ……………………………………..1  ኩበት…………………………………….….2  ከሰል………………………………………..3  ቡታ ጋዝ……………………………………..4  ኤሌክትሪክሲቲ……………………….……….5  ሌላ (ይገለጽ)_________________________6 |  |  |
|  | አሁን፤ ቤተሰቡ ምግብ ለማብሰል በዋነኛነት የሚጠቀመው ምንድን ነው? | እንጨት ……………………………………..1  ኩበት…………………………………….….2  ከሰል………………………………………..3  ቡታ ጋዝ……………………………………..4  ኤሌክትሪክሲቲ……………………….……….5  ሌላ (ይገለጽ)_________________________6 |  |  |
|  | ልጁ ሲወለድ፤ ቤተሰቡ ከሚከተሉት ውስጥ የትኞቹ ነበሩት? | አዎ/የለም አዎ ከሆነ፤ ቁጥር  ብስክሌት _______ ________  ሞቶር ሳይክል ________ ________  መኪና _______ ________  ላም/በሬ _______ ________  የጋማ ከብቶች _______ ________  በግ/ፍየል _______ ________  ዶሮ _______ ________ |  |  |
|  | አሁን፤ ቤተሰቡ ከሚከተሉት ውስጥ የትኞቹ አሉት? | አዎ/የለም አዎ ከሆነ፤ ቁጥር  ብስክሌት _______ ________  ሞቶር ሳይክል ________ ________  መኪና _______ ________  ላም/በሬ _______ ________  የጋማ ከብቶች _______ ________  በግ/ፍየል _______ ________  ዶሮ _______ ________ |  |  |

**ክፍል 3፡ የቤተሰቡ የመጠጥ ውሃና የጽዳት ሁኔታ**

|  | ልጁ ሲወለድ፤ የቤተሰቡ የመጠጥ ውሃ ምንጭ የነበረው? | ቧንቧ……………………………………………...1  የተጠበቀ ምንጭ/ጉድጓድ………………………..…2  ያልተጠበቀ ምንጭ/ጉድጓድ ……………….………3  ወንዝ/ኩሬ……………………………………..…..4  ሌላ (ይገለጽ)_____________________________5 | 304  304 |  |
| --- | --- | --- | --- | --- |
|  | ቤተሰቡ የመጠጥ ውሃ ያገኝ የነበረው ካልተጠበቀ ምንጭ/ጉድጓድ፣ወንዝ/ኩሬ ከሆነ፤ ውሃው ይታከም ነበር? | አዎ…………….…………………………..….….1  የለም…………………………………………..….2 | 304 |  |
|  | ለጥ302 መልስ አዎ ከሆን፤ እንዴት ነበር የሚታከመው? | በማፍላት……………………………….…………1  ኬሚካል በመጨመር…………………………..…2  በአሸዋ በማጠራት……………………….……….3  ሌላ (ይገለጽ)_____________________________4 |  |  |
|  | አሁን፤ የቤተሰቡ የመጠጥ ውሃ ምንጭ ምንድን ነው? | ቧንቧ………………………………………….…...1  የተጠበቀ ምንጭ/ጉድጓድ………………………..…2  ያልተጠበቀ ምንጭ/ጉድጓድ …………………….…3  ወንዝ/ኩሬ…………………………………….…..4  ሌላ (ይገለጽ)____________________________5 | 307  307 |  |
|  | ቤተሰቡ የመጠጥ ውሃ የሚያገኘው ካልተጠበቀ ምንጭ/ጉድጓድ፣ወንዝ/ኩሬ ከሆነ፤ ውሃው ይታከማል? | አዎ…………….……………………………..….1  የለም…………………………………………….2 | 307 |  |
|  | ለጥ305 መልስ አዎ ከሆን፤ እንዴት ነው የሚታከመው? | በማፍላት………………………………….………1  ኬሚካል በመጨመር……………………….………2  በአሸዋ በማጠራት………………………………….3  ሌላ (ይገለጽ)____________________________4 |  |  |
|  | ልጁ ሲወለድ፤ ቤተሰቡ መጸዳጃ ቤት ነበረው? | አዎ…………….……………………..………….1  የለም……………………………………….…….2 | 310 |  |
|  | ለጥ307 መልስ አዎ ከሆነ፤ ምን አይነት ነበር? | ደረቅ መጸዳጃ(simple pit)……………………..…1  ሽታ አልባ(VIP)………………………………....2  በውሃ የሚሰራ……………………………….…..3  ሌላ (ይገለጽ)___________________________4 |  |  |
|  | መጸዳጃ ቤቱ የጋራ ነበር ወይንስ የግል? | የጋራ……………………………………...…..1  የግል……………………………………….…2  ሌላ (ይገለጽ)__________________________3 |  |  |
|  | አሁን ቤተሰቡ መጸዳጃ ቤት አለው? | አዎ…………….……………………………….1  የለም…………………………………….…..….2 | 401 |  |
|  | ለጥ310 መልስ አዎ ከሆነ፤ ምን አይነት ነው? | ደረቅ መጸዳጃ(simple pit)………………….……1  ሽታ አልባ(VIP)………………………………..2  በውሃ የሚሰራ……………………………...…..3  ሌላ (ይገለጽ)__________________________4 |  |  |
|  | መጸዳጃ ቤቱ የጋራ ነበር ወይንስ የግል? | የጋራ……………………………………...1  የግል…………………………………...…2  ሌላ (ይገለጽ)_______________________3 |  |  |
|  | የመጸዳጃ ቤቱ የጽዳት ሁኔታ (ይመልከቱ) | ንጹህ…………………………………………1  ንጹህ ያልሆነ ………………………………….2 |  |  |

**ክፍል 4፡ የናትየዋ የስነ ተዋልዶ ሁኔታ**

|  | ከዚህ ልጅ በፊት እናትየዋ አርግዛ ታውቅ ነበር? | አዎ…………….……………………………..….1  የለም…………………………………………….2 | 412 |  |
| --- | --- | --- | --- | --- |
|  | ለጥ401 መልስ አዎ ከሆነ፤ ስንት ጊዜ አርግዛ ነበር? | __________________በቁጥር |  |  |
|  | ከነዚህ እርግዝናዎች ውስጥ ምንያህሉ ውርጃ፤ ሞቶ የተወለደ፤ በህይወት የተወለደ ነበር? | ቁጥር  ውርጃ፤ __________________________  ሞቶ የተወለደ______________________  በህይወት የተወለደ ____________________  አላውቅም_________________________ |  |  |
|  | ከዚህ ልጅ ወዲያው ቀድሞ የተረገዘው እርግዝና ውጤቱ ምን ነበር ? | ውርጃ……………………………….…….1  ሞቶ የተወለደ…………….……………..…..2  በሂወት የተወለደ ………………….…..…….3  አላውቅም………………………………….99 |  |  |
|  | ከዚህ ልጅ ወዲያው ቀደሞ የነበረው እርግዝና ከተቋረጠ ከስንት ጊዜ በኋላ ነበር ይህ ልጅ የተወለደው? | ____________________ወር |  |  |
|  | ከዚህ ልጅ ወዲያው ቀደሞ የነበረው እርግዝና ከተቋረጠ ከስንት ጊዜ በኋላ ነበር ይህ ልጅ የተረገዘው? | ____________________ወር |  |  |
|  | ለጥ404 መልስ በህይወት ያልተወለደ ከሆነ፤ ከዚህ ልጅ በፊት በህይወት የተወለደው ልጅ ከተወለደ ከስንት ጊዜ በኋላ ነበር ይህ ልጅ የተወለደው? | ____________________ወር |  |  |
|  | ከዚህ ልጅ በፊት የተወለደና የሞተ ልጅ አለ? | አዎ…………….……………………..……..….1  የለም……………………………………..….….2 | 410 |  |
|  | ለጥ408 አዎ ከሆነ፤ ስንት ልጅ ሞቷል? | _______________________በቀጥር |  |  |
|  | ይህ ልጅ ስንተኛ ልጅ ነው? | የመጀመሪያ……………………….……………..…1  ሁለተኛ ……………………………………..…….2  ሶስተኛ ………………………………....................3  አራተኛና ከዚያ በላይ ………………………..……4  አላውቅም ……………………..…………….....99 | 412 |  |
|  | ይህ ልጅ የመጀመሪያ ልጅ ካልሆነ፤ ይህ ልጅ ከመወለዱ በፊት እናትዮዋ ስንት ልጀ ነበራት? | _______________________በቁጥር |  |  |
|  | ይህ ልጅ ከተወለደ በኋላ ሌላ ልጅ ተወልዷል? | አዎ…………….………………………..……….1  የለም…………………………………………….2 | 417 |  |
|  | ለጥ412 መልስ አዎ ከሆነ፤ ስንት ልጅ ተወልዷል? | _______________________በቁጥር |  |  |
|  | ይህ ልጅ ከተወለደ ከስንት ጊዜ በኋላ ነበር ቀጥሎ ያለው ልጅ የተወለደው? | ______________ወር |  |  |
|  | ከዚህ ልጅ በኋላ የተወለደና የሞተ ልጅ አለ? | አዎ…………….……………………………….1  የለም…………………………………….…..….2 | 417 |  |
|  | ለጥ415 አዎ ከሆነ፤ ስንት ልጅ ሞቷል? | _______________________በቁጥር |  |  |
|  | አሁን እናትየዋ ስንት ልጆች አሉዋት? | _______________________በቁጥር |  |  |
|  | እናትየዋ አሁን እርጉዝ ነች? | አዎ …………………….………………….…... 1  የለም ……………………………………..….… 2  እርግጠኛ አይደለሁም…………………………….3 | 501  501 |  |
|  | አዎ ከሆን፤ የስንት ወር እርጉዝ ነች? | __________________ወር |  |  |

**ክፍል 5፡ የጤና አገልግሎት አጠቃቀምን በተመለከተ**

|  | እናትየዋ ይህ ልጅ እርጉዝ እያለች የቅድመ ወሊድ ክትትል (የእርግዝና ምርመራ) ነበራት? | አዎ …………………………………….…...…...1  የለም ………………………………………….2  አላውቅም ……………………………………..99 | 506  506 |  |
| --- | --- | --- | --- | --- |
|  | አዎ ከሆነ፤ የት ነበር የተከታተለችው? | ሆስፒታል…………………………….….…......1  ጤና ጣቢያ……………………………….……2  ጤና ኬላ…………………..….……………….3  ሌላ (ይገለጽ)….. …... ………………………..4  አላውቅም ………………………………..…..99 |  |  |
|  | እርግዝናው ስንት ወር ሲሆነው ነበር ክትትሉን የጀመረችው? | በ3 ወር ጊዜ ውስጥ…………..……..…...1  በ6 ወር ጊዜ ውስጥ……………….……..2  ከ6 ወር በኋላ……….……….…….…...3  አላውቅም……………………….……..99 |  |  |
|  | ለዚህ እርግዝና ብቻ ስንት ጊዜ ነበር ክትትል ያደረገችው? | ____________________በቁጥር  አላውቅም…………………………….99 |  |  |
|  | ክትትል ያደረገላት ማን ነበር? | ሀኪም/ጤና መኮንን………………………..…....1  ነርስ/አዋላጅ ነርስ……………………….…..……...2  ጤና ኤክስቴንሽን ሰራተኛ……………………..…3  የሰለጠነች የልምድ አዋላጅ……………………….4  ያልሰለጠነች የልምድ አዋላጅ………..………….... 5  ሌላ (ይገለጽ)….. …... ………………….…….....6  አላውቅም …………………..………………….99 |  |  |
|  | በዚህ እርግዝና ወቅት እናትየዋ የቴታነስ (ቲቲ) ክትባት ወስዳ ነበር? | አዎ ……………………………….……………...1  የለም …………………………………….…….2  አላውቅም ……………………………………..99 | 508  508 |  |
|  | በዚህ ልጅ እርግዝና ወቅት፤ ለመጨረሻ የወሰደችው ስንተኛው የቲቲ ክትባት ነበር?  (ከካርድ ወይንም ከታሪክ) | ቲቲ1…………………………….….…....1  ቲቲ2…………………………….……....2  ቲቲ3…………………………….……....3  ቲቲ4…………………………….……....4  ቲቲ5…………………………….……....5  አላውቅም…………………………….… 99 |  |  |
|  | ልጁ የት ነበር የተወለደው? | ቤት ………………………………….………...1  ሆስፒታል………………………………..…....…2  ጤና ጣቢያ…………………………………..…3  ጤና ኬላ…………………..…………….…..…4  ሌላ ጤና ደርጅት …... ……………………...…..5  ሌላ (ይገለጽ)….. …... ………………………....6  አላውቅም ………………………..………........99 |  |  |
|  | ልጁ ሲወለድ ማን ነበር ያዋለደው? | የሰለጠነ የጤና ባለሙያ………………………… 1  ጤና ኤክስቴንሽ ሰራተኛ……………………......…2  የሰለጠነች የልምድ አዋላጅ……………………..….3  ያልሰለጠነች የልምድ አዋላጅ………………….….. 4  ዘመድ……………………………………...…...5  ጎረቤት………………………………….....……6  እናትየዋ በራሷ ………….……….................…..7  ሌላ (ይገለጽ)….. …... ……………………..…..8  አላውቅም …………………………….....…......99 |  |  |
|  | ልጁ ሲወለድ እንዴት ነበር? | አንድ……………………..…………..….1  ሁለት መንታ ……………………………..2  ሶስት መንታ ………………………….…..3  አላውቅም ………………………..……...99 |  |  |
|  | ልጁ ሲወለድ ክብደቱ እንዴት ነበር? | በጣም ትልቅ……………………………….1  ትልቅ………………..……………...…..…2  መካከለኛ………………………..….….….3  ትንሽ…………..………………………….4  በጣም ትንሽ……………..…………..……..5  አላውቅም ………………………….……...99 |  |  |
|  | ልጁ ከተወለደ በኋላ፤ የልጁም ሆነ የናትየዋ የጤና ሁኔታ ምርመራ ተደርጎላቸው ነበር? | አዎ…………….…………………….………….1  የለም………………………………………...….2 | 516 |  |
|  | አዎ ከሆነ፤ ምርመራውን ያካሄደው ማን ነበር ? | የሰለጠነ የጤና ባለሙያ………………………..... 1  ጤና ኤክስቴንሽ ሰራተኛ……………………….…2  የሰለጠነች የልምድ አዋላጅ…………………….….3  ያልሰለጠነች የልምድ አዋላጅ…………..……..…... 4  ዘመድ………………………………...........…..5  ጎረቤት………………………………….…..…6  እናትየዋ በራሷ ………….……………........…..7  ሌላ (ይገለጽ)….. …... ……………………..…..8  አላውቅም ………………………..…………....99 |  |  |
|  | ምርመራው የት ነበር የተካሄደው? | ሆስፒታል………………………………….............1  ጤና ጣቢያ……………………..………….…..…2  ጤና ኬላ…………………..……………………..3  ቤት…………………..…………..…………..…4  ሌላ (ይገለጽ)….. …... ……………….……..…....5  አላውቅም ………………………..….................99 |  |  |
|  | ምርመራው ለመጀመሪ ጊዜ የተካሄደው ልጁ ከተወለደ ከስንት ጊዜ በኋላ ነበር? | ____________________ሰዓት  ____________________ቀን  ____________________ሳምንት |  |  |
|  | ልጁ በተወለደ 42 ቀን ውስጥ ስንት ጊዜ ምረመራ ተካሄዶ ነበር? | አንድ ጊዜ……………………………………..1  ሁለት ጊዜ…………………………………….2  ሶስት ጊዜ……………………………….….…3  ከሶስት ጊዜ በላይ………………………..……..4  አላውቅም ……………………….…………..99 |  |  |
|  | ይህ ልጅ ተከትቦ ያውቀል? | አዎ…………….…………………….…..……….1  የለም………………………………….………….2 | 519 |  |
|  | ለጥ517 መልስ አዎ ከሆነ፤ የትኞቹን ክትባቶች ተከትቧል? (ከካርድ/ ከታሪክ) | አዎ የለም  ፖሊዮ0 ______ ______  ቢሲጂ ______ ______  ፖሊዮ1 ______ ______  ፖሊዮ2 ______ ______  ፖሊዮ3 ______ ______  ፔንታ1 ______ ______  ፔንታ2 ______ ______  ፔንታ3 ______ ______  ኩፍኝ ______ ______ |  |  |
|  | የልጁ የክትባት ደረጃ ምን ላይ ነው? (ከካርድ ወይንም ከታሪክ) | ክትባት ጨርሷል……………..………….………1  በከፊል ተከትቧል…………….……………..……2  ምንም አልተከተበም………………………………3  አላውቅም……………………………….….….99 |  |  |
|  | ይህ ልጅ ቫታሚን ኤ (ይህን የሚመስል ወስዷል)? (ከካርድ ወይንም ከታሪክ) | አዎ ……………………………….……….…....1  የለም ………………………………….……..….2  አላውቅም ……………………………………..99 | 521  521 |  |
|  | ለጥ520 አዎ ከሆነ፤ ስንት ጊዜ ወስዷል? | _______________________በቁጥር |  |  |
|  | በህመም ጊዜ ቤተሰቡ በዋናነት ህክምና የሚያገኘው ከየት ነው? | አዎ አይ አላውቅም  ቤት ውስጥ … …………….. 1 2 8 የባህል ሀኪም……..….......... 1 2 8 የመንግስት ጤና ኬላ.……..........1 2 8  የመንግስት ጤና ጣቢያ.................1 2 8  የመንግሰት ሆሰፒታል ……...….1 2 8 የግል ክሊኒክ …..…………….1 2 8 የግል ሆስፒታል…………...… 1 2 8  የግል ፋርማሲ ………………..1 2 8  ሌላ (ይገለጽ)_______________________ |  |  |
|  | እናትዮዋ የቆየ የጤና ችግር ነበረባት? | አዎ ……………………………….………...…...1  የለም ……………………………….….…..….2  ለመመለስ ፍቀደኛ አይደሉም……………….…..99 | 601  601 |  |
|  | ለጥ523 መልስ አዎ ከሆነ፤ ምን አይነት የጤና ችግር?  (ከአንድ በላይ መልስ ይቻላል) | የስኳር ህመም …………………..…………....1  የልብ ህምም………………………………… 2  የደም ግፊት ………………………..…….…..3  የኩላሊት በሽታ ……………………..……….4  ሌላ (ይገለጽ)_________________________5 |  |  |

**ክፍል6፡ ጡት ማጥባትና የልጆች አመጋገብን በተመለከተ**

|  | ልጁ ጡት ጠብቶ ያውቃል? | አዎ ……………………………….………..….....1  የለም ……………………………….………….2 | 604 |  |
| --- | --- | --- | --- | --- |
|  | ልጁ ከተወለደ ከስንት ሰዓት በኋላ ነበር ጡት መጥባት የጀመረው? | _________ሰዓት  _________ቀን |  |  |
|  | በጠቅላለው ለምን ያህል ጊዜ ነበር ጡት የጠባው? | አሁንም ጡት እየጠባነው………………………..1  ለ__________ወር |  |  |
|  | ልጁ በተወለደ በ3 ቀናት ውስጥ ከጡት ውጪ ሌላ ነገር እንዲበላ ወይንም እንዲጠጣ ተሰጥቶት ነበር? | አዎ ……………………………….………...…...1  የለም ………………………………….……...….2  ለመመለስ ፍቀደኛ አይደሉም……………….…..99 | 606  606 |  |
|  | ለጥ604 መልስ አዎ ከሆነ፤ ምን ነበር የተሰጠው? | ወተት (ከእናት ጡት ወተት ውጭ) . . …..…….….1  ውኃ . . . . . . . . ………………………….......2  የስኳር፣ የጨውና ውሀ ውህድ . . . . . ……………3  የፍራፍሬ ጭማቂ . . . . . . . ………………..……4  ለህጻናት የተዘጋጁ ምግቦች. ……………….……..5  ማር . . . . . . . . . . . . …………….……….…..6  ቅቤ . . . . . . …………………………………7  ሌላ (ይገለጽ)__________________________8 |  |  |
|  | ልጁ በተወለደ በ6 ወር ጊዜ ውስጥ ከጡት ውጪ ሌላ ነገር እንዲበላ ወይንም እንዲጠጣ ተሰጥቶት ነበር? | አዎ ………………………………………….…...1  የለም ……………………………….………….2  ለመመለስ ፍቀደኛ አይደሉም…………………...99 | 609  609 |  |
|  | ለጥ606 መልስ አዎ ከሆነ፤ ምን ነበር የተሰጠው? | ውሀ . . . . . . . . …………….……………..…..1  ወተት (ከእናት ጡት ወተት ውጭ) . . . ….…… ….2  ሙቅ………………… . . . . . ………...…...…3  ቅቤ . . . . . . …………………..…….……..…4  የስኳር፣ የጨውና ውሀ ውህድ. . . ... ………..……5  የፍራፍሬ ጭማቂ . . . . . . . ………………..……6  ለህጻናት የተዘጋጁ ምግቦች. ……………….……..7  ሻይ. . . . . . . . . . . . …………………………..8  ማር. . . . . . . . . . . . …………………..….…..9  የአዋቂ ምግብ…………………………………10  ሌላ (ይገለጽ)_________________________11 |  |  |
|  | ልጁ ከተወለደ ከስንት ጊዜ በኋላ ነበር የተጀመረለት? | _____________ቀን  ____________ወር |  |  |
|  | ልጁ በጡጦ ተጠቅሞ ያውቃል? | አዎ ……………………………….…..…..……...1  የለም ……………………………………….….2  ለመመለስ ፍቀደኛ አይደሉም…………………..99 |  |  |
|  | ልጁ እድሜው ከ6ወር በላይ ከሆነው፤ ተጨማሪ ምግብ ተጀምሮለታል? | አዎ …………………………………….………...1  የለም ………………………………….……...….2  ለመመለስ ፍቀደኛ አይደሉም……………….…..99 | 613  613 |  |
|  | ተጨማሪ ምግቡ የተጀመረው ልጁ እድሜው ስንት ሲሆነው ነበር? | _____________ቀን  ____________ወር |  |  |
|  | የተሰጠው ተጨማሪ ምግብ ምንድ ነው? | የላም ወተት…………………………………1  ሙቅ……………………………….…..…..2  የአዋቂ ምግብ………………………….….…3  ሌላ (ይገለጽ)________________________4 |  |  |
|  | ልጁ ከተውለደ ከምን ያህል ጊዜ በኋላ ነበር ገላውን የታጠበው? | ወዲያው እንደተወለደ…………………….……....1  ከአንድ ሰዓት በኋላ………………………..…..….2  ከ6 ሰዓት በኋላ………………………………..…3  ከ12 ሰዓት በኋላ…………………………….……4  ከ24 ሰዓት በኋላ…………………………………5  ከ48 ሰዓት በኋላ…………………………………6 |  |  |
|  | ልጁ ከተወለደ በኋላ የእምብርት ቁስሉ ላይ የተደረገለት ነገር ነበር? | አዎ ……………………………….…….…….....1  የለም …………………………………………….2  ለመመለስ ፍቀደኛ አይደሉም…………………...99 | 616  116 |  |
|  | ለጥ614 መልስ አዎ ከሆነ፤ ምን ነበር የተደረገለት? | ቅቤ………………………………………1  የከብት አዛባ……………………….………2  ሌላ (ይገለጽ)________________________3 |  |  |
|  | ልጁ ታሞ ያውቃል? | አዎ ……………………………….……….…...1  የለም ……………………………….………….2  ለመመለስ ፍቀደኛ አይደሉም……………….…..99 | 619  619 |  |
|  | ለጥ616 መልስ አዎ ከሆነ፤ ለህመሙ ህክምና አግኝቷል/ታክሞ ያውቃል? | አዎ ……………………………….……………...1  የለም …………………………………….…….2  ለመመለስ ፍቀደኛ አይደሉም…………………...99 | 619  619 |  |
|  | አዎ ከሆነ፤ ህክምናውን የት ነበር ያገኘው ? | ቤት……………………………….….....….1  የባህል ህክምና ………………………….....…2  ጤና ኬላ ………………………….…………3  ጤና ጣቢያ…………………….……………..4  ሆስፒታል………………………….……..…..5  የግል ክልኒክ…………….…….…………..…..6  የግል ፋርማሲ………………………………….7  ሌላ (ይገለጽ)…………………………….……8 |  |  |
|  | ልጁ ከተወለደ በኋላ ከሚከተሉት የትኞቹ ተደርገውለታል? | አዎ የለም  የእንጥል መቁረጥ 1 2  የጥርስ መንቀል 1 2  የሰውነት መበሳት/መተኮስ 1 2  ሌላ (ይገለጽ)________________________ |  |  |

**ክፍል 7፡ የሴቶች እኩልነትን በተመለከተ**

|  | ልጁ ሲወለድ፤ እናትየው በመስራትም ሆነ እቃዎችን በመሸጥ ገንዘብ/ገቢ ታገኝ ነበር? | አዎ ………………………………………….…...1  የለም ……………………………….………….2  ለመመለስ ፍቀደኛ አይደሉም…………………...99 | 703  703 |  |
| --- | --- | --- | --- | --- |
|  | ልጁ ሲወለድ፤ ያገኘችውን ገንዘብ/ገቢ ወጪ ማድረግን በተመለከተ የሚወስነው ማን ነበር? | እናትየዋ………………………………….…1  ባልየው…………………………………….2  ሁለቱ በጋራ…………………………………3  ሌላ (ይገለጽ)________________________4 |  |  |
|  | አሁን፤ እናትየዋ በመስራትም ሆነ እቃዎችን በመሸጥ ገንዘብ/ገቢ ታገኛለች? | አዎ ……………………………………..…..…....1  የለም ………………………………….…….....2  ለመመለስ ፍቀደኛ አይደሉም……………….…..99 | 705  705 |  |
|  | አሁን፤ ያገኘችውን ገንዘብ/ገቢ ወጪ ማድረግን በተመለከተ የሚወስነው ማን ነው? | እናትየዋ……………………………..….…1  ባልየው……………………………..….….2  ሁለቱ በጋራ…………………………….….3  ሌላ (ይገለጽ)_______________________4 |  |  |
|  | ልጁ ሲወለድ፤ የባልየው ገቢ ወጪ ማድረግን በተመለከተ የሚወስነው ማን ነበር? | እናትየዋ………………………….…….…1  ባልየው………………………….…….….2  ሁለቱ በጋራ………………………….……3  ባልየው ገቢ የለውም……………………..…4  ሌላ (ይገለጽ)_______________________5 |  |  |
|  | አሁን፤ የባልየው ገቢ ወጪ ማድረግን በተመለከተ የሚወስነው ማን ነው? | እናትየዋ……………………………..….…1  ባልየው…………………………….…..….2  ሁለቱ በጋራ…………………..……………3  ባልየው ገቢ የለውም………………...………4  ሌላ (ይገለጽ)_______________________5 |  |  |
|  | ልጁ ሲወለድ፤ የናትየውን የጤና አገልግሎት አጠቃቀምን በተመለከተ የሚወስነው ማን ነበር? | እናትየዋ………………………………...…1  ባልየው………………………………..…..2  ሁለቱ በጋራ………………………….….…3  ሌላ ሰው………………………………..…4  ሌላ (ይገለጽ)_______________________5 |  |  |
|  | አሁን፤ የናትየውን የጤና አገልግሎት አጠቃቀምን በተመለከተ የሚወስነው ማን ነው? | እናትየዋ……………………………..……1  ባልየው……………………….…….…….2  ሁለቱ በጋራ……………………….………3  ሌላ ሰው………………………….………4  ሌላ (ይገለጽ)_______________________5 |  |  |
|  | ልጁ ሲወለድ፤ ቤት ውስጥ ወሳኝ የሆኑ ዕቃዎች ግዢን በተመለከተ የሚወስነው ማን ነበር? | እናትየዋ…………………………………………1  ባልየው……………………………………….….2  ሁለቱ በጋራ……………………….……………3  ሌላ ሰው……………….………………………4  ሌላ (ይገለጽ)____________________________5 |  |  |
|  | አሁን፤ ቤት ውስጥ ወሳኝ የሆኑ ዕቃዎች ግዢን በተመለከተ የሚወስነው ማን ነው? | እናትየዋ…………………………….……1  ባልየው…………………………….…….2  ሁለቱ በጋራ………………………………3  ሌላ ሰው…………………………………4  ሌላ (ይገለጽ)_______________________5 |  |  |
|  | ልጁ ሲወለድ፤ የእናትየዋ የቤተሰብም ሆነ የዘመድ ጥየቃ በተመለከተ የሚወስነው ማን ነበር? | እናትየዋ…………………………..………1  ባልየው…………………………….….….2  ሁለቱ በጋራ…………………….…………3  ሌላ ሰው…………………………….……4  ሌላ (ይገለጽ)_______________________5 |  |  |
|  | አሁን፤ የእናትየዋ የቤተሰብም ሆነ የዘመድ ጥየቃ በተመለከተ የሚወስነው ማን ነው? | እናትየዋ……………………………..……1  ባልየው……………………………..…….2  ሁለቱ በጋራ……………………….………3  ሌላ ሰው……………………….…………4  ሌላ (ይገለጽ)_______________________5 |  |  |
|  | ልጁ ሲወለድ፤ ባልየው የቤት ስራዎችን (ልጅ መጠበቅ፣ ምግብ ማብሰል የመሳሰሉትን) በመስራት እናትየዋን ያግዛት ነበር? | አዎ ……………………………….………...1  የለም ………………………………….…….2  ለመመለስ ፍቀደኛ አይደሉም…………………..99 |  |  |
|  | አሁን፤ ባልየው የቤት ስራዎችን (ልጅ መጠበቅ፣ ምግብ ማብሰል የመሳሰሉትን) በመስራት እናትዋን ያግዛታል? | አዎ ……………………………….………...1  የለም ………………………………….…….2  ለመመለስ ፍቀደኛ አይደሉም…………………..99 |  |  |
|  | በእርሶ አመለካከት፤ ከሚከተሉት በየትኞቹ ሁኔታዎች ባል ሚስቱን ቢመታት ችግር የለውም ይላሉ?  ለሱ ሳትነግረው ለጉዳይዋ ከቤት ከወጣች?  ልጆችን በደንብ ካልተንከባከበች?  እሱን ከተሟገተችው?  ለግብረስጋ ጥያቄው እምቢተኛ ከሆነች?  ምግብ ካሳረረች? | አይ የለም አላውቅም  ከቤት መውታት. . . . . . .…..1 2 8  ልጅ አለመንከባከብ. . ……...1 2 8  መሟገት . . . . . . . . . . . ….1 2 8  ለግብረስጋ አለመስማማት.. . .1 2 8  ምግብ ማቀጠል . . . . . . . ....1 2 8 |  |  |
|  | ልጁ ሲወለድ፤ ለጥ715 ያለዎት ምላሽ የተለየ ነበር? | አዎ ……………………………….……….…....1  የለም ………………………………….……..….2 | 738 |  |
|  | ለጥ 716 መልስ አዎ ከሆነ፤ በትኛው ሁኔታ? | አይ የለም አላውቅም  ከቤት መውታት. . . . . . .…..1 2 8  ልጅ አለመንከባከብ. . …..… 1 2 8  መሟገት . . . . . . . . . . . …..1 2 8  ለግብረስጋ አለመስማማት.. . . 1 2 8  ምግብ ማቀጠል . . . . . . . ... 1 2 8 |  |  |
|  | ልጁ ሲወለድ፡ ባልዎ ደብድበዎት ያውቃል? | አዎ ……………………………….………...1  የለም ………………………………….…….2  ለመመለስ ፍቀደኛ አይደሉም……………….…..99 |  |  |
|  | አሁንስ፤ ባልዎ ደብድበዎት ያውቃል? | አዎ ……………………………….………...1  የለም ………………………………….…….2  ለመመለስ ፍቀደኛ አይደሉም…………………..99 |  |  |
|  | ኢትዮጵያ ውስጥ፤ አንድ ባል ሚስቱን እንዳይደበድብ የሚከለክል ህግ አለ እንዴ? | አዎ ……………………………….………...1  የለም ………………………………….…….2  ለመመለስ ፍቀደኛ አይደሉም…………………..99 |  |  |

ጥያቄዎቼን ጨርሻለሁ

በጣም አድርጌ አመሰግናለሁ

**በመረጃ ተቆጣጣሪው ተረጋግጧ**

ስም____________________ ፊርማ _____________ ቀን_______________
